# Antibacterial and Antifungal Activities of Spices

**DOI:** 10.3390/ijms18061283

**Published:** 2017-06-16

**Authors:** Qing Liu, Xiao Meng, Ya Li, Cai-Ning Zhao, Guo-Yi Tang, Hua-Bin Li

**Affiliations:** 1 Guangdong Provincial Key Laboratory of Food, Nutrition and Health, Department of Nutrition, School of Public Health, Sun Yat-sen University, Guangzhou 510080, China; liuq248@mail2.sysu.edu.cn (Q.L.); mengx7@mail2.sysu.edu.cn (X.M.); liya28@mail2.sysu.edu.cn (Y.L.); zhaocn@mail2.sysu.edu.cn (C.-N.Z.); tanggy5@mail2.sysu.edu.cn (G.-Y.T.); 2 South China Sea Bioresource Exploitation and Utilization Collaborative Innovation Center, Sun Yat-sen University, Guangzhou 510006, China

**Keywords:** spice, antibacterial activity, antifungal effect, antimicrobial property, essential oil, clove, oregano, thyme

## Abstract

Infectious diseases caused by pathogens and food poisoning caused by spoilage microorganisms are threatening human health all over the world. The efficacies of some antimicrobial agents, which are currently used to extend shelf-life and increase the safety of food products in food industry and to inhibit disease-causing microorganisms in medicine, have been weakened by microbial resistance. Therefore, new antimicrobial agents that could overcome this resistance need to be discovered. Many spices—such as clove, oregano, thyme, cinnamon, and cumin—possessed significant antibacterial and antifungal activities against food spoilage bacteria like *Bacillus subtilis* and *Pseudomonas fluorescens*, pathogens like *Staphylococcus aureus* and *Vibrio parahaemolyticus*, harmful fungi like *Aspergillus flavus*, even antibiotic resistant microorganisms such as methicillin resistant *Staphylococcus aureus*. Therefore, spices have a great potential to be developed as new and safe antimicrobial agents. This review summarizes scientific studies on the antibacterial and antifungal activities of several spices and their derivatives.

## 1. Introduction

Microbial pathogens in food may cause spoilage and contribute to foodborne disease incidence, and the emergence of multidrug resistant and disinfectant resistant bacteria—such as *Staphylococcus aureus* (*S. aureus*), *Escherichia coli* (*E. coli*), and *Pseudomonas aeruginosa* (*P. aeruginosa*)—has increased rapidly, causing the increase of morbidity and mortality [1]. Weak acids such as benzoic and sorbic acids [2], which are commonly applied in food industry as chemical preservatives to increase the safety and stability of manufactured foods on its whole shelf-life by controlling pathogenic and spoilage food-related microorganisms [3], can result in the development of microbiological resistance [4]. Moreover, chemical preservatives cannot completely eliminate several pathogenic bacteria like *Listeria monocytogenes* (*L. monocytogenes*) in food products or delay the growth of spoilage microorganisms [5]. Natural products, as substitutes of synthetic chemical preservatives, are increasingly accepted because they are innately better tolerated in human body and have inherent superiorities for food industry [4]. The antimicrobial activities of natural products are necessary to be studied and applied in food industry.

Morbidity and mortality are mainly caused by infectious diseases all over the world. The World Health Organization reported that 55 million people died worldwide in 2011, with one-third of the deaths owing to infectious diseases [6]. Antibiotic resistant microorganisms can increase mortality rates because they can survive and recover through their ability to acquire and transmit resistance after exposure to antibiotic drugs, which are one of the therapies to infectious diseases [7]. Antibiotic resistant bacteria threaten the antibiotic effectiveness and limit the therapeutic options even for common infections [8]. The decline in research and development of new antibacterial agents, which are able to inhibit antibiotic resistant disease-causing microorganisms such as *S. aureus*, aggravates the emerging antibiotic resistance [9]. Therefore, much attention should be paid to natural products, which could be used as effective drugs to treat human diseases, with high efficacy against pathogens and negligible side effects.

Spices have been used as food and flavoring since ancient times [10], and as medicine and food preservatives in recent decades [11,12]. Many spices—such as clove, oregano, thyme, cinnamon, and cumin—have been applied to treat infectious diseases or protect food because they were experimentally proved to possess antimicrobial activities against pathogenic and spoilage fungi and bacteria [10,13,14]. Moreover, the secondary metabolites of these spices are known as antimicrobial agents, the majority of which are generally recognized as safe materials for food with insignificant adverse effects [11]. Therefore, spices could be candidates to discover and develop new antimicrobial agents against foodborne and human pathogens.

This review summarizes the scientific studies on the antibacterial and antifungal activities of spices and their derivatives, and some suggestions and prospects are offered for future studies.

## 2. Clove

Clove (*Eugenia caryohyllata*), belonging to family Myrtaceae, is widely used in medicine as antiseptic against infectious diseases like periodontal disease due to the antimicrobial activities against oral bacteria [15]. Clove is also commonly applied in food industry as a natural additive or antiseptic to increase shelf-life due to the effective antimicrobial activities against some foodborne pathogens [16]. The main active component of clove oil and extract was found, i.e., eugenol [15,17].

### 2.1. Antimicrobial Activities of Clove

Antimicrobial activities of clove water extract were studied in vitro and in vivo against pathogenic microorganisms (*S*. *aureus* and *E*. *coli*, in a model of pyelonephritis) [18]. An in vitro study was conducted with the agar well diffusion method, and the results suggested that clove water extract showed antibacterial activity against *S*. *aureus* (minimum inhibitory concentration (MIC): 2 mg/mL) and *E*. *coli* (MIC: 2.5 mg/mL). While in vivo, the study was conducted in 40 adult male albino rats, and the results confirmed the efficacy of clove extract as natural antimicrobials. The direct antimicrobial activities of ultra-fine powders of ball-milled cinnamon and clove were tested by Kuang et al. [19] against *E*. *coli*, *S*. *aureus*, *Brochothrix thermosphacta* (*B. thermosphacta*), *Lactobacillus rhamnosus* (*L. rhamnosus*), and *Pseudomonas fluorescens* (*P. fluorescens*) from meat, using broth dilution method. Clove powder showed strong inhibitory effects on five microorganisms tested with the MICs ranging from 1.0% *w*/*v* (*L*. *rhamnosus* and *B*. *thermosphacta* ) to 2.0% *w*/*v* (*P*. *fluorescens*), and the inhibitory effects were positively associated with the concentrations of powder, which increased from 0.5% to 2.5% *w*/*v*.

Clove could destroy cell walls and membranes of microorganisms, and permeate the cytoplasmic membranes or enter the cells, then inhibit the normal synthesis of DNA and proteins [16]. Eugenol, the major component of clove, could inhibit the production of amylase and proteases in *Bacillus cereus* (*B. cereus*) and has the ability of cell wall deterioration and cell lysis [20].

### 2.2. Comparison of Antimicrobial Activities of Clove and Other Spices

Badei et al. [21] tested the antimicrobial activities of cardamom, cinnamon and clove essential oils (EOs) against nine Gram-positive bacterial strains, four Gram-negative bacterial strains, seven molds, and two yeasts, compared with phenol, using the disc diffusion method. Clove EO showed the highest antimicrobial activity, and the antimicrobial spectra (diameter of inhibition zones) of 10% clove EO was 1.48 times as that of 10% phenol. Schmidt et al. [22] evaluated the antifungal effects of eugenol-containing EOs of 4 spices on 38 *Candida albicans* (*C. albicans*) isolates, of which 12 were isolated from oropharynges, 16 from vaginas, and 10 from damaged skin, using the microdilution method. Clove EO possessed the strongest antifungal activities against all *C*. *albicans* strains among the tested spices. Pure eugenol alone exhibited weaker antifungal activities than clove leaf EO. Angienda et al. [23] investigated the antimicrobial activities of EOs of four spices against *Salmonella typhimurium* (*S. typhimurium*), *E*. *coli*, *B. cereus*, and *Listeria innocua* (*L. innocua*) by agar diffusion test. Clove EO showed the most effective inhibition against both Gram-positive bacteria and Gram-negative bacteria compared with three other EOs, with the MICs ranging from 1.25% *v*/*v* (*B. cereus*) to 2.50% *v*/*v* (*S*. *typhimurium* and *E*. *coli*). Lomarat et al. [17] reported the antimicrobial activities of EOs from nine spices against histamine-producing bacteria including *Morganella morganii* (*M*. *morganii*), by determining MICs and minimum bactericidal concentrations (MBCs) using the broth dilution assay, and also found the antibacterial compounds of EOs by bioautography-guided isolation. The results indicated that the clove EO was the most effective against *M*. *morganii* among nine tested spices with MIC 0.13% *v*/*v* and MBC 0.25% *v*/*v*. The eugenol was identified as the active component of clove EO by thin layer chromatography bioautography assay.

Shan et al. [24] tested the antibacterial activities of ethanol extracts from five spices and herbs against *L*. *monocytogenes*, *S*. *aureus*, and *Salmonella enterica* (*S. enterica*) in raw pork by counting bacterial enumeration. When treated with clove extract, raw pork samples were found with the fewest colonies of tested bacteria. Bayoub et al. [25] reported the antimicrobial activities of ethanol extracts of 13 plants including clove against *L. monocytogenes*, the MICs were determined by agar well diffusion test. The results showed that clove extract was the most effective inhibitor against *L*. *monocytogenes* compared with the other 12 selected plant ethanol extracts, with the MIC 0.24 mg/mL. Cui et al. [26] tested the antimicrobial activities of 90 plant extracts (water and 99.5% ethanol extracts) against *Clostridium* spp. Clove water extract was found with the greatest antimicrobial activity against *Clostridium botulinum* in trypticase peptone glucose yeast extract broth (pH = 7.0) among all the water extracts, and the MICs of clove extract ranged from 0.1% to 0.2% against *Clostridium* spp. Antimicrobial effects of 3 extracts (ethyl acetate, acetone, and methanol extracts) of 12 plants were tested on 2 fungi (*Kluyveromyces marxianus* (*K. marxianus*) and *Rhodotorula rubra* (*R. rubra*)) and 8 bacteria (*Klebsiella pneumoniae* (*K*. *pneumoniae*), *Bacillus megaterium* (*B. megaterium*), *P. aeruginosa*, *S*. *aureus*, *E*. *coli*, *Enterobacter cloacae* (*E. cloacae*), *Corynebacterium xerosis* (*C. xerosis*), and *Streptococcus faecalis* (*S. faecalis*)) by the disc diffusion method [27]. Clove exhibited the most effective inhibitory impacts. The methanol extract from clove showed inhibition against microorganisms (diameter of inhibition zones (DIZs): 8–24 mm) tested except *K*. *pneumoniae*. The acetone extract showed inhibition against microorganisms (DIZs: 8–18 mm) tested except *R*. *rubra* and *K*. *pneumoniae*. The ethyl acetate extract only showed antibacterial activity against *B*. *megaterium* (DIZ: 7 mm). Liang et al. [28] observed the antimicrobial activities of seven spices, and different concentrations of extracts and EOs in each spice were used to test the effects on the growth of spoilage microorganisms in apple cider by total plate counts. Clove products showed the strongest antimicrobial activities compared with other spices tested. Nearly seven log reduction of microorganisms was observed at 0.8% *v*/*v* in the cider for clove oil and 2% *w*/*w* for clove powder at room temperature. Badhe et al. [29] tested the antimicrobial activities of many spice and herb powders against *S*. *aureus*, *S. typhimurium*, *E*. *coli*, and *B*. *cereus* at refrigerated temperature (8 ± 2 °C) for intervals of 0, 3, 6, 12, 24, and 48 h. The results indicated that at the concentration of 2%, clove powder showed highest effect on *S*. *aureus* followed by *E*.*coli* and *S*. *typhimurium*, and at 24 h under refrigeration, clove powder led to a significant reduction of bacteria counting.

### 2.3. The Application of Clove as Antimicrobial Agents in Food Packaging

Clove EO and its functional extracts have been incorporated into films, the antimicrobial activities of which have been evaluated in some studies. In a study, chitosan at high, moderate and low molecular mass were elaborated with antimicrobial films which were incorporated with EOs and extracts from two spices [30]. Then the antimicrobial effects of the films were investigated on *E*. *coli*, *S*. *typhimurium*, *S*. *aureus*, *B*. *cereus*, and *L*. *monocytogenes*. The films prepared by low molecular mass chitosan with 2% EO and ethyl heptanoate extract from clove showed antimicrobial activities against a majority of the tested strains. In another study, the researchers tested the antimicrobial activities of EOs and functional extracts of cumin, clove, and elecampane against *E*. *coli*, *S. typhimurium*, *B*. *cereus*, *S*. *aureus*, and *L*. *monocytogenes* by determining the MICs and MBCs [31]. They also evaluated the antibacterial activities of edible films prepared by EOs and functional extracts of spices based on chitosan polymeric structure against the same bacteria by determining the DIZs. Clove EO showed the best inhibitory effects with the MIC of 500 mg/L on all the bacteria tested, clove extracts showed very similar MICs to those of EO, except ethyl caproate extract of clove against *L*. *monocytogenes* (MIC of 750 mg/L) and ethyl heptanoate extract of clove against *B*. *cereus* (MIC of 250 mg/L). Among the chitosan films added with EOs, only clove showed inhibition zones of all tested bacteria except *L*. *monocytogenes*. The ethyl heptanoate extract of clove film also possessed antibacterial activities against all tested bacteria, weaker than those of clove EO though. Liu et al. [32] evaluated the antimicrobial activities of spice EOs against microbial populations in chilled pork stored in PE film antimicrobial package using the disk diffusion method to determine the DIZs and serial dilution assay to determine the MICs. Clove EO was the most effective against microorganisms tested among all the spice EOs tested. The MICs of clove EO were 0.10%, 0.10%, and 0.30% *v*/*v* against *Enterobacteriaceae*, *S*. *aureus*, and *Pseudomonas* sp., respectively. Spice EOs possessed the ability to decrease the number of spoilage populations, but not the species diversity of spoilage microbiota.

Collectively, clove EO and extracts could prevent against some food spoilage and foodborne pathogens (Table 1), especially Gram-positive bacteria. The MICs of clove were less than 2.5% against tested microorganisms like *P*. *fluorescens*, *S. typhimurium*, *E*. *coli*, *B. cereus*, and *L. innocua.* Generally speaking, the qualities of the papers cited are good and the results are reliable.

## 3. Oregano

Oregano (*Origanum vulgare*), belonging to family Lamiaceae, has been used as food seasoning and flavoring for a long time. The major components associated with antimicrobial activities in oregano EO were proved to be carvacrol and thymol [33].

### 3.1. Antimicrobial Activities of Oregano

Babacan et al. [34] evaluated the antimicrobial activities of oregano extract against various *Salmonella* serotypes by evaluating the bacterial growth with disc diffusion method. The results showed that DIZs of oregano were 15, 19, and 16 mm for *Salmonella gallinarum* (*S. gallinarum*), *Salmonella enteritidis* (*S. enteritidis*), and *S. typhimurium*, respectively. Santoyo et al. [35] observed the antimicrobial activities of EO-rich fractions of oregano which were selectively precipitated in the second separator in different conditions against six microorganism strains (*S*. *aureus*, *Bacillus subtilis* (*B*. *subtilis*), *E*. *coli*, *P*. *aeruginosa*, *C*. *albicans*, and *Aspergillus niger* (*A. niger*)), using the disk diffusion and broth dilution methods. The results showed that all of the supercritical fluid extraction fractions exhibited antimicrobial effects on tested microorganisms, and the most efficient fraction was obtained with 7% ethanol at 150 bar and 40 °C. De Souza et al. [36] evaluated the effects of heating (at the temperatures of 60, 80, 100, and 120 °C, at a duration of 1 h for each) on the antimicrobial activities of oregano EO against 9 microorganism strains (*C*. *albicans*, *Candida krusei* (*C*. *krusei*), *Candida tropicalis* (*C*. *tropicalis*), *B*. *cereus*, *E*. *coli*, *S*. *aureus*, *Yersinia enterocolitica* (*Y. enterocolitica*), *S*. *enterica*, and *Serratia marcescens* (*S. marcescens*)), using the solid medium diffusion procedure. The results indicated that heating treatment showed no significant effects on the antimicrobial activities of EO, with the DIZs and MICs of heated EO close to those of EO kept at room temperature (MICs ranging from 10 to 40 μL/mL).

Oregano could bind to sterols in the fungal membranes of *C. albicans* strains [37], but the exact mechanisms of action on other microorganisms are to be further studied. Carvacrol, one of the major components of oregano, could interact with cell membranes through changing the permeability for small cations [38]. As the chemical compounds in EO and extracts of oregano are complex, they could inhibit microorganisms through different cell targets.

### 3.2. Comparison of Antimicrobial Activities of Oregano and Other Spices

Ozcan et al. [39] investigated the antifungal activities of four spice decoctions against six molds (*Fusarium oxysporum* f. sp. *phaseoli*, *Macrophomina phaseoli* (*M. phaseoli*), *Botrytis cinerea* (*B. cinerea*), *Rhizoctonia solani* (*R. solani*), *Alternaria solani* (*A. solani*), and *Alternaria parasiticus* (*A. parasiticus*)). The results showed that the mycelial growth were 100% inhibited by 10% oregano decoction in culture medium. Ai-Turki et al. [40] tested the antimicrobial activities of aqueous extracts of four plants against *E*. *coli* and *B*. *subtilis* using the disc diffusion method. Oregano extract showed the best antibacterial effects on two bacteria compared with three other spice extracts, and *B*. *subtilis* showed more sensitivity than *E*. *coli*. Marques et al. [41] assessed the antimicrobial activities of the EOs of oregano and marjoram against *S*. *aureus* isolated from poultry meat using the disk diffusion method, and the MICs and MBCs were tested using the microdilution technique. All the *S. aureus* strains were susceptible to oregano EO with the MICs ranging from 6.25 to 25 μL/mL, but four of the isolates were resistant to ampicillin and one was resistant to tetracycline. Bozin et al. [42] investigated the antimicrobial activities of 3 spice EOs against 13 bacterial strains using the hole-plate agar diffusion method and 6 fungi by the microdilution technique. The results indicated that the most effective antibacterial activities were expressed by oregano EO, even on multiresistant strains of *P. aeruginosa* and *E*. *coli*. Viuda-Martos et al. [43] studied the antimicrobial activities of EOs from six spices against six bacteria (*Lactobacillus curvatus* (*L. curvatus*), *Lactobacillus sakei* (*L. sakei*), *Staphylococcus carnosus* (*S. carnosus*), *Staphylococcus xylosus* (*S. xylosus*), *Enterobacter gergoviae* (*E. gergoviae*) and *Enterobacter amnigenus* (*E. amnigenus*)), using the disc diffusion method. Oregano EO was the most effective against bacteria tested, with DIZs ranging from 35.29 mm (*S. xylosus*) to 57.90 mm (*E. amnigenus*). Santurio et al. [44] reported the antimicrobial activities of EOs of eight spices against *E*. *coli* strains isolated from poultry and cattle faeces by determining the MICs using the broth microdilution technique. The results showed that the most effective against all *E*. *coli* strains in the study was oregano EO. Khosravi et al. [45] investigated the antifungal activities of *Artemisia sieberi* and oregano EOs against *Candida glabrata* (*C. glabrata*) isolated from patients with vulvovaginal candidiasis by determining the MICs and minimal fungicidal concentrations (MFCs), using the broth macrodilution method. The results indicated that the EOs inhibited all tested *C*. *glabrata* isolates concentration-dependently, with the MICs ranging from 0.5 to 1100 μg/mL (mean: 340.2 μg/mL) for oregano. Dal Pozzo et al. [46] studied the antimicrobial activities of 7 spice EOs, and some majority constituents of these spices such as carvacrol, thymol, cinnamaldehyde, and cineole against 33 *Staphylococcus* spp. isolates from herds of dairy goats, by determining the MICs and MBCs using the broth microdilution method. Oregano and thyme possessed equally strong antimicrobial activities among EOs. Santos et al. [47] evaluated the antimicrobial activities of four spices against several bacteria like *S. aureus* and *E*. *coli* isolated from vongole and bacteria standard ATCC (American Type Culture Collection): *E*. *coli*, *S. aureus*, and *Salmonella choleraesuis* (*S. choleraesuis*), by determining the MICs using diffusion test. Oregano and clove EOs presented antimicrobial activities against all tested bacteria, but oregano presented larger DIZs of 26.7 mm (*E*. *coli*) and 29.3 mm (*S. aureus*). Hyun et al. [48] tested the antibacterial effects of various spice EOs including oregano on total mesophilic microorganisms in products (fresh leaf lettuce and radish sprouts) using the dipping method. One species of oregano (in the USA) EO showed the best effects on maintaining reduced levels of total mesophilic microorganisms in fresh leaf lettuce and radish sprouts compared with the control.

### 3.3. The Application of Oregano as Antimicrobial Agents in Food Packaging

The antimicrobial effects of pure EOs of four spices and chitosan-EOs films on *L*. *monocytogenes* and *E*. *coli* were evaluated in vitro by agar diffusion test [49]. The antimicrobial activities of EOs alone and incorporated in the films were similar following the order: oregano >> coriander > basil > anise. When used in inoculated bologna samples at 10 °C and stored for five days, pure chitosan films led to 2 log reduction of *L*. *monocytogenes*, 3.6–4 log reduction of *L*. *monocytogenes*, and 3 log reduction of *E*. *coli* were observed in films incorporated with 1% and 2% oregano EO.

All the above studies are of good quality, and oregano showed strong antimicrobial activities against microorganism strains such as *Staphylococcus* spp. and *S. aureus* isolates with larger DIZs and lower MICs, MBCs, and MFCs compared with several other spices (Table 2). Future studies could focus on the application of oregano and its EO in food industry, and also the possible mode of action.

## 4. Thyme

Thyme (*Thymus vulgaris*), belonging to family Lamiaceae, is a subshrub native to the western Mediterranean region. Thyme is widely used as a spice to add special flavor to foods. In recent studies, thyme was found to possess efficient antimicrobial activities and was used in some foods to extend the shelf-life [50].

### 4.1. Antimicrobial Activities of Thyme

A study evaluated the antimicrobial activities of thyme EO against bacteria (*B. subtilis*, *S. aureus*, *Staphylococcus epidermidis* (*S. epidermidis*), *P. aeruginosa*, *E*. *coli*, and *Mycobacterium smegmatis* (*M. smegmatis*)) and fungal strains (*C. albicans* and *Candida vaginalis*) [51]. Thyme EO showed effective bactericidal and antifungal activities against tested microorganism strains with MICs ranging from 75 to 1100 μg/mL for bacteria, and from 80 to 97 μg/mL for fungi. In another study, EOs obtained from thyme harvested at four ontogenetic stages were tested for their antibacterial activities against nine strains of Gram-negative bacteria and six strains of Gram-positive bacteria using the bioimpedance method to test the bacteriostatic activities and plate counting technique to study the inhibitory effects by direct contact [52]. The results indicated that all the thyme EOs had significant bacteriostatic activities against the microorganisms tested. Furthermore, the antimicrobial activities of EOs of four *Thymus* species (*T. vulgaris*, *T. serpyllum*, *T. pulegioides*, and *T. glabrescens*) were determined by agar diffusion method [53]. *T. vulgaris* and *T. serpyllum* EOs were the most efficient as they inhibited all the tested bacteria (*P. aeruginosa*, *Cronobacter sakazakii* (*C*. *sakazakii*), *L. innocua*, and *Streptococcus pyogenes* (*S*. *pyogenes*)) and yeasts (*C. albicans* and *Saccharomyces cerevisiae* (*S. cerevisiae*)) both at original and half-diluted concentrations. *P*. *aeruginosa*, *L*. *innocua*, and *S*. *pyogenes* were highly and equally sensitive to the *Thymus* oils, while *C*. *sakazakii* exhibited limited sensitivity, and the sensitivity of the two yeast strains were similar to that of *C*. *sakazakii*, but *S*. *cerevisiae* was a little more sensitive than *C*. *albicans*.

The major active compound of thyme is thymol, which exerted its antimicrobial action through binding to membrane proteins by hydrophobic bonding and hydrogen bonding, and then changing the permeability of the membranes [20]. Thymol also decreased intracellular adenosine triphosphate (ATP) content of *E. coli* and increased extracellular ATP, which could disrupt the function of plasma membranes [54]. As thymol was proved to act differently against Gram-positive and Gram-negative bacteria [20], the exact mechanisms of antimicrobial action should be further studied.

### 4.2. Comparison of Antimicrobial Activities of Thyme and Other Spices

Al-Turki et al. [55] reported the antibacterial activities of thyme, peppermint, sage, black pepper and garlic hydrosols against *B. subtilis* and *S. enteritidis*, using the agar disk diffusion method. Thyme hydrosol demonstrated more significant inhibitory effects on *B. subtilis* and *S. enteritidis* than sage, peppermint, and black pepper hydrosols, with the mean DIZs 20 mm for *B. subtilis* and 15 mm for *S. enteritidis*. According to another study, the antimicrobial effects of the six plant hydrosols on *S. aureus*, *E*. *coli*, *S*. *typhimurium*, *P*. *aerugenosa*, and *C. albicans* were tested by determining the microbial growth zones on hydrosol agar plates and control agar plates [56]. The results showed that at 15% thyme hydrosol completely inhibited *E*. *coli* and *S*. *typhimurium*, but *C. albicans* was inactive to the tested hydrosols. Girova et al. [57] assessed the antimicrobial activities of five plant EOs against psychrotrophic microorganisms (*P. fluorescens*, *Pseudomonas putida* (*P*. *putida*), *P*. *fragi*, *B. thermosphacta*, and *C. albicans*) isolated from spoiled chilled meat products and some reference strains (*P*. *fluorescens* ATCC 17397, *P*. *putida* NBIMCC (National Bank for Industrial Microorganisms and Cell Cultures) 561, *P*. *aeruginosa* ATCC 9027, and *C*. *albicans* ATCC 10231) using the method of disc diffusion and serial broth dilution. The results indicated that the antimicrobial effects of the EOs were equal at 37 °C and 4 °C. Thyme EO exhibited the highest antimicrobial activities with the MICs ranging from 0.05% to 0.8% *w*/*v*. Hajlaoui et al. [58] observed the anti-*Vibrio alginolyticus* (*V. alginolyticus*) activities of five aromatic plant EOs using agar well diffusion test, and the MICs and MBCs were examined using the broth microdilution susceptibility method. Thyme EO was proved to be the most efficient against 13 *V. alginolyticus* strains compared with 4 other EOs, with the MICs ranges of 0.078–0.31 mg/mL and MBCs ranges of 0.31–1.25 mg/mL. Also, Viuda-Martos et al. [59] assessed the growth inhibition of some indicators of spoilage bacteria strains (*L*. *innocua*, *S*. *marcescens*, and *P*. *fluorescens*) and the concentration effects of five spice EOs using the agar disc diffusion method. Only the EO of thyme showed inhibitive effects on all tested bacteria at all added doses (100%, 50%, 25%, 12.5%, and 5%). Aliakbarlu et al. [60] evaluated the antibacterial activities of EOs from thyme, *Thymus kotschyanus*, *Ziziphora tenuior*, and *Ziziphora clinopodioides*, against two Gram-positive bacteria (*B. cereus* and *L. monocytogenes*) and two Gram-negative bacteria (*S. typhimurium* and *E. coli*), using the agar disc diffusion and micro-well dilution assay. The EO of thyme showed the highest antibacterial activities, with the widest inhibition zones and the lowest MICs (0.312–1.25 μL/mL), and *B. cereus* was the most sensitive bacterium tested. Hyun et al. [48] investigated the antibacterial effects of several EOs on 18 pathogenic bacteria and 15 spoilage bacteria by agar disc diffusion test. The results showed that thyme-1 (*T. vulgaris*) EO and thyme-2 (*T. vulgaris* ct *linalool*) EO exerted the highest antibacterial activities against 18 pathogenic bacteria strains compared with other spices, except for *P. aeruginosa*. Thyme-1 EO also demonstrated the best antibacterial effects on spoilage bacteria. In addition, the antimicrobial effects of 17 spices and herbs against *Shigella* strains were tested in another study [61]. The MICs were determined by the agar dilution method with dried ground spices and herbs added to the broth and agar, and the results showed that MICs of thyme were 0.5–1% *w*/*v* for the *Shigella* strains. The study also used various combinations of temperatures (12, 22, and 37 °C), pH values (5.0, 5.5, and 6.0), and NaCl concentrations (1%, 2%, 3%, and 4% *w*/*v*), and the inclusion or exclusion of thyme or basil at 1% *w*/*v* in a Mueller–Hinton agar model system to test the inhibitory effects of thyme and basil. In the presence of thyme, *Shigella flexneri* (*S. flexneri*) did not develop Colony-Forming Units (CFU) during the seven-day incubation period for 16 of the 18 tested combinations.

Some studies compared the antimicrobial activities of different extracts of thyme. Martins et al. [62] evaluated and compared the antimicrobial activities of the infusion, decoction, and hydroalcoholic extracts prepared from thyme against *S. aureus*, *S. epidermidis*, *E. coli*, *Klebsiella* spp., *P. aeruginosa*, *Enterobacter aerogenes* (*E. aerogenes*), *Proteus vulgaris* (*P. vulgaris*), and *Enterobacter sakazakii* (*E. sakazakii*) using the disc diffusion halo test. For Gram-positive species, thyme extracts only presented activity against *S*. *epidermidis*, and hydroalcoholic extract showed a lower antibacterial activity than decoction and infusion extracts, which showed the similar activities. For Gram-negative species, thyme extracts showed antimicrobial activities in the order of *E*. *coli* > *P*. *vulgaris*, *P*. *aeruginosa* > *E*. *aerogenes = E*. *sakazakii*; decoction and hydroalcoholic extracts had similar effects against the bacteria except *P*. *aeruginosa*, while the lowest activity was observed in infusion extracts. Moreover, the antifungal effects of thyme EO, hydrosol and propolis extracts on natural mycobiota on the surface of sucuk were evaluated [63]. The results showed that potassium sorbate (15% *w*/*v*, in water), thyme EO (10 mg/mL, in dimethyl sulfoxide), and propolis extract (50 mg/mL, in dimethyl sulfoxide) reduced by 4.88, 2.45, and 2.05 log CFU/g in yeast-mold counting compared with sterile water, respectively.

Aman et al. [64] analyzed the polyphenolic fractions and oil fractions of oilseeds from 4 spices, including thyme, for their antimicrobial activities against 35 bacterial strains. The results showed that oil fractions of all spice oilseeds were more active than their polyphenolic fractions, and thyme oil fraction had the highest antibacterial activities compared with other spice oilseeds. Aznar et al. [65] studied the growth of *Candida lusitaniae* (*C. lusitaniae*) on different concentrations of nisin (0.1–3 mmol/L), thymol (0.02–1.5 mmol/L), carvacrol (0.02–1 mmol/L), or cymene (0.02–3 mmol/L) in broths (pH = 5, 25 °C), and also evaluated the inhibitory activity of thymol against *C. lusitaniae* in tomato juice. Thymol, carvacrol, and cymene totally inhibited the yeast growth for more than 21 days at 25 °C when the concentrations were higher than 1 mmol/L. Compared with the control without thymol, the activity of thymol against *C*. *lusitaniae* in tomato juice was significant.

In conclusion, the results obtained from a number of investigations with good quality indicated that thyme possessed effective antimicrobial activities against several pathogenic and spoilage bacteria and fungi, like *S. aureus* and *E*. *coli*, with low MICs (≤1100 μL/mL) (Table 3).

## 5. Cinnamon

Cinnamon (*Cinnamomum zeylanicum*), belonging to family Lauraceae, is widely applied in savory dishes, pickles, and soups [66]. Cinnamaldehyde, cinnamyl acetate, and cinnamyl alcohol are the three main compounds of cinnamon [67]. Due to its antimicrobial activities, cinnamon is also used in cosmetics or food products [11], and also used as health-promoting agents to treat diseases like inflammation, gastrointestinal disorders, and urinary infections [68,69].

### 5.1. Antimicrobial Activities of Cinnamon

The antimicrobial activities of cinnamon were evaluated in some studies. Gupta et al. [70] compared the antimicrobial activities of cinnamon extract (50% ethanol) and EO against 10 bacteria and 7 fungi by the agar well diffusion method. Cinnamon EO was more effective than cinnamon extract against tested microorganisms, with the MICs ranging from 1.25% to 5% *v*/*v*. Cinnamon EO exerted the strongest effect on *B*. *cereus* among bacteria, and *Rhizomucor* sp. among fungi. Cinnamon extract showed the highest activities against *B*. *cereus* among bacteria, and *Penicillium* sp. among fungi. Ceylan et al. [71] tested the antibacterial effects of cinnamon, sodium benzoate, potassium sorbate, and their combinations on *E*. *coli* at 8 and 25 °C in apple juice. The results showed that 0.3% *w*/*v* cinnamon provided 1.6 log CFU/mL reduction on *E*. *coli* at 8 °C and 2.0 log CFU/mL reduction at 25 °C. Cinnamon had synergistic effects with sodium benzoate and potassium sorbate on *E*. *coli* at 8 and 25 °C. Recently, the anti-biofilm effects of cinnamon EO and liposome-encapsulated cinnamon EO on methicillin resistant *S. aureus* (MRSA) were evaluated in a study by scanning electron microscopy and laser scanning confocal microscopy analyses [72]. Cinnamon EO possessed effective antibacterial activity and prominent anti-biofilm activity against MRSA. In the presence of liposomes, the stability and the acting time of cinnamon EO were further improved.

The major component of cinnamon, cinnamaldehyde, possesses antimicrobial effects on microorganisms, as it inhibited cell wall biosynthesis, membrane function, and specific enzyme activities. More specific cellular targets of cinnamaldehyde are still required to be studied in detail [73].

### 5.2. Comparison of Antimicrobial Activities of Cinnamon and Other Spices

Mvuemba et al. [74] evaluated the inhibitory effects of aqueous extracts of four spices (cinnamon, ginger, nutmeg, and horseradish) on the growth of mycelial of various spoilage pathogens (*A. niger*, *Fusarium sambucinum* (*F. sambucinum*), *Pythium sulcatum* (*P*. *sulcatum*), and *Rhizopus stolonifera* (*R*. *stolonifera*)). At the concentration of 0.05 g/mL, cinnamon extract totally inhibited *A*. *niger* and *P*. *sulcatum*, while at the level of 0.10 and 0.15 g/mL *F*. *sambucinum* and *R*. *stolonifer* were completely inhibited, respectively. Another study conducted by Wang et al. [75] tested the antibacterial effects of five plant aqueous extracts on five bacteria (*S. aureus*, *Lactobacillus* sp., *B. thermosphacta*, *Pseudomonas* spp., and *E*. *coli*) by the aerobic plate count method and disc diffusion. Cinnamon aqueous extract was the only one to inhibit all the tested microorganisms at the concentration of 1% *w*/*v*. The inhibitory effects were stronger with the increase of extract concentrations from 1% to 5% *w*/*v*. In the same way, the antimicrobial activities of the hydrosols of six spices (basil, clove, cardamom, cinnamon, mustard, and thyme) against five microorganisms (*S. aureus*, *E*. *coli*, *S. typhimurium*, *P. aeruginosa*, and *C*. *albicans*) were tested [56]. The inhibition percentage of cinnamon hydrosol was 10–33.8% at 5% *v*/*v* hydrosol, 10–66.5% at 10% *v*/*v* hydrosol, and 10–100% at 15% *v*/*v* hydrosol against microorganisms tested except *C*. *albicans*. Moreover, *S. aureus* was the most sensitive strain to cinnamon hydrosol, while *P*. *aeruginosa* was the least sensitive strain. Agaoglu et al. [76] examined the antimicrobial activities of diethyl ether extracts of six spices used in meat products against eight strains of bacteria (*S. aureus*, *K. pneumonia*e, *P. aeruginosa*, *E*. *coli*, *Enterococcus faecalis* (*E. faecalis*), *M. smegmatis*, *Micrococcus luteus* (*M. luteus*), and *C. albicans*), by the disc diffusion. Among all the spices tested, only cinnamon exerted inhibitory activities against all the tested microorganisms. *S. aureus* and *C*. *albicans* were the most sensitive to cinnamon, while *E*. *coli* was the least. Keskin et al. [27] investigated the antimicrobial effects of the ethyl acetate, acetone, and methanol extracts of 12 plant species on 8 bacterial and 2 fungi species using the disc assay. Cinnamon methanol extract exerted antimicrobial effects on all tested microorganisms, while the ethyl acetate extract showed inhibition against tested microorganisms except *P*. *aeruginosa* and *R*. *rubra*, and the acetone extract showed inhibition against tested microorganisms except *R*. *rubra*. Revati et al. [77] explored the antimicrobial activities of seven Indian spice ethanol extracts against *Enterococci* (including 215 enterococcal strains) isolated from human clinical samples with the agar well diffusion method. Crude ethanol extract of cinnamon was the most effective against all the clinical isolates of *Enterococci*, with the DIZs ranging from 31 to 34 mm. Moreover, the antimicrobial activities of 8 spice EOs against 6 bacterial species and 10 fungal species were tested in a study using the disk diffusion assay and MICs were determined using the agar dilution test [78]. Cinnamon EO possessed the strongest inhibition effects on all tested microorganisms among all spices examined with the MICs ranges of 0.015–2.0 mg/mL. Compared with bacteria, fungi were more sensitive to cinnamon EO.

Collectively, all the mentioned studies with good quality demonstrated that cinnamon showed antimicrobial activities covering a wide range of species, such as MRSA and *A*. *niger*, at low MICs (Table 4), indicating that cinnamon had great potential to provide health benefits through application in food industry.

## 6. Cumin

Cumin (*Cuminum cyminum*) is an aromatic plant belonging to the Apiaceae family. Cumin has been used since ancient time as an ingredient in foods in Middle East, and cumin seeds have long been used as antiseptic and disinfectant in India [80]. Cuminaldehyde, cymene, and terpenoids are the major bioactive constituents of cumin EOs [81].

### 6.1. Antimicrobial Activities of Cumin

In a study, the antimicrobial activities of cumin EO against *E*. *coli*, *S. aureus*, *S. faecalis*, *P*. *aeruginosa*, and *K. pneumoniae* were investigated by agar diffusion and dilution methods [81]. *E*. *coli*, *S. aureus*, and *S*. *faecalis* were susceptive to various cumin EO dilutions while *P*. *aeruginosa* and *K*. *pneumoniae* were resistant. In another study, the antifungal activities of cumin seeds EO against 1230 fungi isolated from food samples were tested [82]. The EO was fungicidal to most of the fungal species, and exerted a broad spectrum of fungal toxicity at MIC (0.6 μL/mL) against all 19 foodborne fungi strains except *R. stolonifer*. Furthermore, Abd El Mageed et al. [83] explored the effects of microwaves on EO of cumin seeds on its antimicrobial activities against *E*. *coli*, *S. aureus*, *P*. *aeruginosa*, *A*. *niger*, *A*. *parasiticus*, and *C*. *albicans* using the disk diffusion method. Both microwave and conventionally (oven) roasted cumin oils had similar antimicrobial effects on microorganisms tested and were more effective than those of raw oils. Reza et al. [80] studied the effects of ã-irradiation (10 and 25 kGy) on the antibacterial activities of cumin against *E*. *coli*, *P*. *aeruginosa*, *B*. *cereus*, and *S. aureus*, by the agar well diffusion method and disk diffusion method. The results indicated that cumin EO exerted antibacterial effects on bacteria tested, and ã-irradiation (10 and 25 kGy) to cumin seeds had no significant effects on the antimicrobial activities of cumin.

### 6.2. Comparison of Antimicrobial Activities of Cumin and Other Spices

Chaudhry et al. [84] determined the antibacterial effects of aqueous infusions and aqueous decoctions of 3 spices on 188 bacteria from 11 genera isolated from oral cavity of apparently healthy individuals, by the disc diffusion test. Aqueous decoction of cumin possessed the highest antimicrobial activities for it showed inhibitory effects on 73% of the bacteria strains tested. Cumin EO was also more effective than some spice EOs as reported. Iacobellis et al. [85] evaluated the antimicrobial activities of EOs of cumin and *Carum carvi* L. against *E. coli* and the genera *Pseudomonas*, *Clavibacter*, *Curtobacterium*, *Rhodococcus*, *Erwinia*, *Xanthomonas*, *Ralstonia*, and *Agrobacterium* using the agar diffusion test. Cumin EO showed antibacterial effects on both Gram-positive and Gram-negative bacteria except *Pseudomonas viridiflava*, which was resistant to 8 μL EO, the highest level tested. Ozcan et al. [86] examined the antimicrobial activities of nine spice EOs at three concentrations (1%, 10%, and 15% *v*/*v*) against *S. typhimurium*, *B. cereus*, *S. aureus*, *E. faecalis*, *E*. *coli*. *Y. enterocolitica*, *S. cerevisiae*, *Candida rugosa*, *Rhizopus oryzae*, and *A. niger*. The results showed that cumin EO was effective against all tested bacterial species as well as *S*. *cerevisiae* and *Candida rugosa* among fungi. Stefanini et al. [87] analyzed the antimicrobial activities of EOs of spices (fennel seeds, dill, cumin, and coriander) by determining the DIZs. The results indicated that cumin was effective against *E*. *coli*, *P*. *aeruginosa*, and *Salmonella* sp. with DIZs of 18, 10, and 23 mm, respectively. In another study, the antimicrobial activities of EOs of six spices against *L. curvatus*, *L. sakei*, *S. carnosus*, *S. xylosus*, *E. gergoviae*, and *E. amnigenus* were assessed using the agar disc diffusion method [43]. Cumin EO was the second effective against bacteria tested with DIZs ranging from 31.23 mm (*L*. *sakei*) to 38.17 mm (*E*. *gergoviae*). Moreover, another study evaluated the antimicrobial activities of EOs of five spices against different microorganism species by the disc diffusion method and discussed the possible effects in vitro between plants and antibiotics [88]. Cumin inhibited all tested bacteria and fungi. The application of cumin with gentamicin, cephalothin, and ceftriaxone showed synergistic effects against *Pseudomonas pyocyaneus* (*P*. *pyocyaneus*) and *Aeromonas hydrophila* (*A. hydrophila*), but showed antagonistic effects against other bacteria tested. Similarly, the possible synergistic interactions of some spice EOs on antibacterial activities against six foodborne bacteria—*B*. *cereus*, *L*. *monocytogenes*, *M. luteus*, *S. aureus*, *E*. *coli*, and *S. typhimurium*—were evaluated by micro broth dilution, checkerboard titration, and time-kill methods [89]. The results showed that coriander and cumin seed oil combination exhibited synergistic interactions on antibacterial activities.

Consequently, cumin had antimicrobial effects on several microorganisms like *E*. *coli*, *S. aureus*, and *S*. *faecalis* at low concentrations (Table 5). In the future, the mechanisms of antimicrobial action of cumin and its major components—cuminaldehyde and cymene—on other microorganisms should be further studied.

## 7. Rosemary

Rosemary (*Rosmarinus officinalis*), belonging to the Lamiaceae family, is a perennial shrub with pleasant smell and grows all over the world. Rosemary has been used in pharmaceutical products and traditional medicine, and also used as a flavoring agent in food products due to its desirable flavor, antioxidant activities, and antimicrobial activities [90,91].

### 7.1. Antimicrobial Activities of Rosemary

Tavassoli et al. [91] reported rosemary EO suppressed *Leuconostoc mesenteroides*, *Lactobacillus delbruekii*, *S. cerevisia*, and *C. krusei*. The results indicated that rosemary EO showed higher inhibitory effects on bacteria (MICs: 0.5–1.5 mg/mL) tested than on yeasts. Bozin et al. [92] identified the antimicrobial activities of EOs of rosemary and sage against 13 bacterial strains and 6 fungi by the microdilution technique. Compared with bifonazole, rosemary EO showed better antifungal activities especially against *C*. *albicans*, *Trichophyton tonsurans* (*T*. *tonsurans*), and *Trichophyton rubrum* at lower MICs (15.0–30.2 μL). Rosemary EO also expressed important antibacterial activities on *E*. *coli*, *S. typhimurium*, *S*. *enteritidis*, and *Shigella sonei*. Weerakkody et al. [93] compared the antibacterial effects of extracts from seven spices and herbs on *E*. *coli*, *S. typhimurium*, *L*. *monocytogenes*, and *S. aureus* by the agar disc diffusion and broth dilution assay. The results of both methods indicated that hexane extract of rosemary exhibited significantly higher antibacterial activities than ethanol and water extracts against all bacteria tested except *S*. *typhimurium* with the MICs ranging from 1.25 to 5.0 mg/mL.

### 7.2. Comparison of Antimicrobial Activities of Rosemary and Other Spices

Additionally, Krajcova et al. [94] observed the antimicrobial activities of five plant ethanol extracts against *B*. *cereus*, *E*. *coli*, *P*. *aeruginosa*, *S. aureus*, and *L*. *monocytogenes* using the dilution method and the description of growth curves of the tested bacteria. Rosemary extract was proved to be the most effective at all concentrations (0.1%, 0.05%, 0.02%, and 0.01% *w*/*w*). At the concentration of 0.01% *w*/*w*, rosemary extract only inhibited *P*. *aeruginosa* and *E*. *coli*, while the higher extract concentrations were effective against all other bacteria. Zhang et al. [95] examined the antimicrobial effects of 14 spice ethanol extracts and their mixtures on *L*. *monocytogenes*, *E*. *coli*, *P*. *fluorescens*, and *L. sake* using the well diffusion test. Individual extract of rosemary showed strong antimicrobial activities, and the combination of rosemary and liquorice extracts showed the best inhibitory effects on all tested microorganisms. Kozlowska et al. [96] tested the antimicrobial activities of aqueous extracts from 5 spices against 8 Gram-positive bacteria and 12 Gram-negative bacteria by the disc diffusion assay. Rosemary exhibited its inhibitory effects with a broader spectrum than the other four spices, as the MICs were 0.125–0.5 mg/mL for all the tested Gram-positive bacteria and 0.25–0.5 mg/mL for four Gram-negative bacteria. Weerakkody et al. [97] studied the antimicrobial activities of two extract combinations against *L*. *monocytogenes* and *S. aureus* and naturally spoilage microflora on instant shrimp stored for 16 days at 4 or 8 °C. Both combinations (galangal, rosemary, and lemon; galangal and rosemary) significantly decreased the levels of aerobic bacteria and lactic acid bacteria, but showed no effects on *L*. *monocytogenes* or *S. aureus*. Azizkhani et al. [90] evaluated the antimicrobial effects of rosemary, mint, and a mixture of tocopherols against microorganisms from the sausages. The application of rosemary significantly inhibited the growth of microorganisms and the lowest microbial counts were obtained in samples containing both rosemary and mint, indicating the possible synergistic effects. Toroglu [88] evaluated the antimicrobial activities of five spice EOs by the disc diffusion method and discussed possible effects of plants and antibiotics. Rosemary had antimicrobial effects on all tested fungi and bacteria. The combination of rosemary EO and cephalothin antibiotics showed synergic effects on *S. aureus*, while the combination of rosemary EO and ceftriaxone antibiotics showed no effect.

Above all, the papers cited are of good quality and indicated that rosemary EO and extracts were found antimicrobial at low MICs against some bacteria and fungi, especially Gram-positive bacteria such as *S. aureus* (Table 6). Some studies indicated that rosemary showed synergic effects with some spices and antibiotics such as galangal and cephalothin. The mechanisms of antimicrobial action of both rosemary and its major components should be further studied.

## 8. Garlic

Garlic (*Allium sativum*) belongs to the Liliaceae family [98]. The antimicrobial activities of garlic have been recognized for many years, and the active component was identified as allicin, a diallyl thiosulfinate (2-propenyl-2-propenethiol sulfonate) [99].

### 8.1. Antimicrobial Activities of Garlic

In a study, Sallam et al. [100] examined the antimicrobial effects of fresh garlic, garlic powder, and garlic oil on microorganisms in raw chicken sausage by aerobic plate count. Garlic materials showed antimicrobial activities in such an order: fresh garlic > garlic powder > garlic oil > butylated hydroxyanisole. Another study also assessed the antimicrobial activities of dried garlic powders made by different drying methods against *S. aureus*, *E*. *coli*, *S*. *typhimurium*, *B*. *cereus* and a mixed lactic culture containing *Lactobacillus delbrueckii* subsp. bulgaricus and *Streptococcus thermophilus* [99]. Fresh garlic exhibited the highest activities followed by freeze-dried powder. The retaining of active components responsible for antimicrobial activities was mainly affected by both drying temperature and time.

Chopped garlic at concentrations from 0% to 10% were investigated for the antimicrobial effects in ground beef (stored at refrigerator and ambient temperatures) and raw meatballs (stored at room temperature) by determining the colony counts of total aerobic mesophilic bacteria, yeast, and molds at 2, 6, 12, and 24 h after storage [101]. The results indicated that chopped garlic delayed the growth of microorganisms in ground meat, which depended on the garlic concentrations. The addition of garlic (5% or 10%) to the raw meatball mix reduced the microorganism counting, in terms of total aerobic mesophilic bacteria, yeast, and mold counts.

Garlic EO penetrated the cellular membranes and even the menbranes of organelles like mitochondria, damaged organelles, and resulted in the death of *C. albicans* [102]. Furthermore, garlic EO induced differential expression of several critical genes including those involved in oxidation-reduction processes, and cellular response to drugs and starvation.

### 8.2. Comparison of Antimicrobial Activities of Garlic and Other Spices

Some studies compared the antimicrobial activities of different spices. Indu et al. [103] studied the antimicrobial effects of 5 spice extracts on 20 serogroups of *E*. *coli*, 8 serotypes of *Salmonella*, *L*. *monocytogenes* and *A. hydrophila* using the agar well method and filter paper method. Garlic extract exhibited significant antibacterial activities at all concentrations (100%, 75%, 50%, and 25%) against all test microorganisms except *L*. *monocytogenes*, and the activity against *E*. *coli* was linearly dependent with concentration. Joe et al. [104] reported the antimicrobial effects of garlic, ginger, and pepper ethanol extracts on *K. pneumoniae*, *S. aureus*, *M. morgani*, *C*. *albicans*, *E*. *coli*, and *P. vulgaris* using the filter paper assay. Garlic extract exerted superior antibacterial activities at all concentrations (1000, 1500, and 2000 ppm), especially against *P*. *vulgaris* and *M*. *morgani*, and the activities were a linear function of concentrations. Geremew et al. [105] examined the antimicrobial activities of six spice crude extracts (acetone, ethanol, and hexane extracts) against *E*. *coli*, *S. aureus*, *S*. *flexneri*, and *Streptococcus pneumoniae* by the agar well diffusion method. Garlic was the most effective against all tested pathogens except *S*. *flexneri*. Among different solvent extracts used, garlic acetone extract exhibited the highest antibacterial activities. Touba et al. [106] tested the antimicrobial activities of crude extracts of seven spices against three Roselle pathogens by poisoned food technique. The results indicated that the cold water extract of garlic exhibited good antifungal activities against all three tested fungi, and hot water extract of garlic showed the best antifungal activities. Nejad et al. [98] reported the antibacterial effect of garlic aqueous extract on *S. aureus* in hamburger. Samples treated with garlic aqueous extract were kept in refrigerator for one and two weeks, and were frozen for one, two, and three months, before being tested by the microbial counts. The first- and second-week samples were significantly reduced by all the 1, 2, and 3-mL extracts, which were added to 100 g hamburger samples, respectively, showing 2 and 3-mL extracts were more effective. In treatment of one, two, and three-month samples, the growth of *S. aureus* was significantly decreased by the 2 and 3-mL extracts. Al-Turki [55] explored the antimicrobial activities of five spice hydrosols (thyme, peppermint, sage, black pepper, and garlic) against *B*. *subtilis* and *S*. *enteritidis* using the agar disk diffusion method. Garlic hydrosol exhibited stronger antibacterial activities against *B*. *subtilis* and *S*. *enteritidis* compared with thyme, peppermint, sage, and black pepper hydrosols.

In conclusion, garlic showed great antimicrobial activities at low concentrations against several pathogenic microorganisms like *E*. *coli* and *S. aureus* (Table 7). Fresh garlic was found to possess higher antimicrobial activities than garlic powder and oil.

## 9. Ginger

Ginger (*Zingiber officinale*), belonging to the family of Zingiberaceae [107], is widely used as an ingredient in food, pharmaceutical, cosmetic, and other industries. Some volatile compounds which are responsible for antimicrobial activities in ginger were á-pinene, borneol, camphene, and linalool [108].

### 9.1. Antimicrobial Activities of Ginger

Ginger was proved to possess antimicrobial activities in several studies. Singh et al. [109] determined the antifungal activities of EO and oleoresin of ginger against *Aspergillus terrus*, *A. niger*, *Aspergillus flavus* (*A. flavus*), *Trichothecium roseum* (*T. roseum*), *Fusarium graminearum* (*F. graminearum*), *F. oxysporum*, *Fusarium oxysporum* (*F. monoliforme*), and *Curvularia palliscens*, by food poison and inverted petri-plate technique. The results showed that the EO 100% inhibited *F. oxysporum*, while the oleoresin 100% inhibited *A. niger*. Park et al. [107] compared the ethanol and *n*-hexane extracts of ginger and five ginger constituents against three anaerobic Gram-negative bacteria, *Porphyromonas gingivalis* (*P. gingivalis*), *Porphyromonas endodontalis*, and *Prevotella intermedia*. The results indicated that ginger extracts exhibited antibacterial activities against three tested bacteria. Two highly alkylated gingerols showed significant inhibition against the growth of these oral pathogens with the MICs ranging from 6 to 30 µg/mL, and also killed the oral pathogens at a MBC range of 4–20 µg/mL. Sa-Nguanpuag et al. [108] evaluated the in vitro and in vivo antimicrobial activities of ginger oils which were obtained by hydrodistillation and solvent extraction method. The results showed that the oils extracted by both methods possessed antimicrobial activities against *B*. *subtilis*, *Bacillus nutto*, *P. aerugenosa*, *Rhodoturola* sp., *Samonella newport*, *S. enteritidis*, and *Fusarium* sp.; except *E. coli*, *Campylobactor coli*, and *Campylobactor jejuni* (*C. jejuni*) in vitro*.* In the case of shredded green papaya, when the package was added with 5 and 10 μL ginger oils the growth of microorganisms was inhibited well, while with 15 μL ginger oil a reduction in growth rate was observed.

### 9.2. Comparison of Antimicrobial Activities of Ginger and Other Spices

Yoo et al. [110] investigated the antibacterial activities of EOs from ginger and mustard against *Vibrio* species at various temperatures. The results indicated that EOs from ginger and mustard could inhibit the growth of *Vibrio parahaemolyticus* and *Vibrio vulnificus* at 5 °C of storage. Indu et al. [103] tested the antibacterial activities of 5 spice extracts against 20 serogroups of *E*. *coli*, 8 serotypes of *Salmonella*, *L*. *monocytogenes*, and *A. hydrophila* by the agar well method and filter paper method. The results indicated that ginger extract possessed inhibitory effects on two serogroups of *E. coli*. Mvuemba et al. [74] assessed the antimicrobial activities of four spice water extracts against the mycelial growth of *A*. *niger*, *F. sambucinum*, *P. sulcatum*, or *R. stolonifera*. The results demonstrated that ginger extract significantly suppressed the mycelial growth of tested microorganisms, and *P*. *sulcatum* was 100% inhibited by 0.05 g/mL of ginger extract. Touba et al. [106] tested the antifungal activities of crude extracts of seven spices made by cold water and hot water against *Phoma exigua* (*P. exigua*), *Fusarium nygamai* (*F. nygamai*), and *R. solani* by poisoned food technique. The results showed that hot water extracts from garlic and ginger possessed the best antifungal activities. Cold water extracts were commonly more effective than hot water extracts on tested pathogens. In another study, the antibacterial activities of 7 ethanol extracts of spices against 215 high levels gentamicin resistant enterococcal strains isolated from clinical samples were evaluated by the well diffusion method [77]. The results indicated that only cinnamon and ginger extracts were found to have activities against all the isolates, with the DIZs of ginger ranged from 27 to 30 mm.

Collectively, ginger was proved to possess significant antimicrobial activities against some common microorganisms such as *P. aerugenosa* both in vivo and in vitro at low concentrations (Table 8). Ginger could also inhibit pathgens like *P. gingivalis* and enterococcal isolates with low MICs and MBCs. The exact mechanisms of action of ginger on bacteria and fungi were rarely studied and need futher exploration.

## 10. Basil

Basil (*Ocimum basilicum*) is one of the oldest spices, which is widely used in the flavoring of confectionary, baked goods, condiments, etc. Basil oil was also used in perfumery, as well as in dental and oral products [112]. Basil is a natural spice which possesses antimicrobial activities as many studies have reported.

### 10.1. Antimicrobial Activities of Basil

In a study, the antimicrobial activities of EOs from aerial parts of basil (collected at full flowering stage during summer, autumn, winter, and spring) against *S. aureus*, *E*. *coli*, *B*. *subtilis*, and *Pasteurella multocida*, as well as pathogenic fungi *A. niger*, *Mucor mucedo*, *Fusarium solani* (*F. solani*), *Botryodiplodia theobromae*, and *R. solani* were assessed by the disc diffusion method and the MICs were determined by a microdilution broth susceptibility assay [113]. The results indicated that basil EOs possessed antimicrobial activities against all tested microorganisms. Antimicrobial activities of the EOs varied significantly as seasons changed, and EOs from winter and autumn crops exhibited greater antimicrobial activities. In another study, the antimicrobial activities of chloroform, acetone and 2 different concentrations of methanol extracts of basil against 10 bacteria and 4 yeasts were determined by the disc diffusion assay [114]. Methanol extracts provided inhibition zones on *P. aeruginosa*, *Shigella* sp., *L*. *monocytogenes*, *S. aureus*, and two strains of *E*. *coli*, but the chloroform and acetone extracts exhibited no effects. Kocic-Tanackov et al. [115] reported the antifungal effects of basil extract on *Fusarium* species (*Fusarium oxysporum*, *Fusarium proliferatum*, *Fusarium subglutinans*, and *Fusarium verticillioides* isolated from cakes), by the agar plate test. Basil extract showed significant activities against *F*. *proliferatum* and *F*. *subglutinans* at the concentration of 0.35 and 0.70% *v*/*v*, but showed lower activities against other tested *Fusarium* species. Basil extract 100% inhibited aerial mycelium of all tested *Fusarium* spp. at 1.50% *v*/*v*. Beatovic et al. [116] investigated the antimicrobial activities of EOs of 12 basil cultivars against 8 bacterial species (*B*. *cereus*, *Micrococcus flavus*, *S. aureus* and *E. faecalis*, *E*. *coli*, *P*. *aeruginosa*, *S*. *typhimurium*, and *L*. *monocytogenes*) and 7 fungi (*Aspergillus fumigatus* (*A. fumigatus*), *A*. *niger*, *Aspergillus versicolor* (*A. versicolor*), *Aspergillus ochraceus* (*A. ochraceus*), *Penicillium funiculosum*, *Penicillium ochrochloron*, and *Trichoderma viride*) by a modified microdilution technique. All basil EOs tested showed significant antimicrobial activities, with MICs ranging from 0.009 to 23.48 μg/mL for bacteria and 0.08–5.00 μg/mL for fungi. All the EOs showed 100-fold higher antibacterial activities than ampicillin for some bacteria, and 10- to 100-fold higher antifungal activities than the commercial antifungal agents, e.g., ketoconazole and bifonazole.

### 10.2. Comparison of Antimicrobial Activities of Basil and Other Spices

El-Habib [117] investigated the antifungal activities of seven spice EOs against *A. flavus* and aflatoxin producted by *A*. *flavus* strain. The results showed that basil EO delayed the growth of *A*. *flavus.* At 150 μL/100 mL, basil EO completely inhibited *A*. *flavus*, and effectively controlled the aflatoxin B1 production. Lomarat et al. [17] tested the antibacterial activities of eight EOs against *M. morganii*, a histidine decarboxylase producing bacteria, by microdilution assay. Basil EO possessed the antibacterial activity against *M*. *morganii* (MIC: 2.39 mg/mL, MBC: 4.77 mg/mL), and the active compound of basil oil was methyl chavicol.

Generally, basil has been proved to possess effects of inhibiting some microorganisms at low MICs especially fungi like *A*. *flavus* (Table 9), but the mechanisms of action have been rarely explored. Therefore, future studies are needed.

## 11. Fennel

Fennel (*Foeniculum vulgare*), belonging to family Umbellifarae [118], is widely planted in temperate zones and the tropical belt for its aromatic fruits, and is used as an ingredient in the cooking [119]. The EO of fennel seeds has been reported with significant antifungal activities and antibacterial activities.

### 11.1. Antimicrobial Activities of Fennel

In a study, the antibacterial activities of fennel seeds EO against *Streptococcus mutans* (*S. mutans*) strains were tested [120]. The results showed that growths of all *S*. *mutans* strains tested were completely inhibited by fennel seeds EOs at concentrations higher than 80 ppm. Diao et al. [119] also determined the antibacterial activities of EO from fennel seeds against several foodborne pathogens by the kill-time curve assay method. The results showed that fennel seeds EO exerted antibacterial effects on *Streptomyces albus* (*S. albus*), *B. subtilis*, *S. typhimurium*, *Shigella dysenteriae* (*S. dysenteriae*). and *E*. *coli*, among which *S*. *dysenteriae* was the most sensitive with the lowest MIC (0.125 mg/mL) and MBC (0.25 mg/mL). In another study, the antimicrobial activities of crude extract of fennel was determined using the agar diffusion method against *E*. *coli*, *S. blanc*, *P. merabilis*, *P. vulgaris*, *S. epidemidis*, *S. saprophyticus*, *A. versicolor*, *A. fumigates*, and *Penicilium camemberti* [121]. The results indicated that the crude extract of fennel had a great potential as an antimicrobial material against all the nine microorganisms tested, especially fungal strains. Some studies also tested the methanol, ethanol, and acetone extracts of fennel. In a study, the antifungal activities of EO and acetone extract of fennel against 10 fungi were assessed by the inverted petriplate method [118]. The results showed that fennel EO completely inhibited *A. niger*, *A. flavus*, *F. graminearum*, and *Fusarium moniliforme* (*F. moniliforme*) at 6 μL (in 20 mL culture medium), and it was effective on *A*. *niger* even at 4 μL.

Fennel seed EO could break the permeability of cell membrane of *S. dysenteriae* and result in the leakage of electrolytes, losses of proteins, reducing sugars, etc., and eventually lead to the decomposition and death of cells [119].

### 11.2. Comparison of Antimicrobial Activities of Fennel and Other Spices

The antimicrobial activities of cumin and fennel EOs on *S. typhimurium* and *E*. *coli* were compared by the disc diffusion method and dilution method [122]. Fennel EO was more effective than cumin EO, with the lowest MICs of 0.031% and 0.062% *v*/*v* against *S*. *typhimurium* and *E*. *coli*, respectively. Nguyen et al. [123] evaluated the antimicrobial activities of methanol and ethanol extracts of eight spices against *B*. *subtilis*, *E. faecalis*, *L*. *innocua*, *E*. *coli*, *P. putida*, *Providencia stuartii*, and *Acetobacter calcoaceticus* (*A. calcoaceticus*) by the Kirby-Bauer disc diffusion method. Methanol and ethanol extracts from fennel seeds exhibited the best antimicrobial effects with the largest DIZs on six out of the seven bacteria except *E*. *coli*.

Fennel EO and extracts were effective against several foodborne pathogens with low MICs and MBCs such as *S*. *dysenteriae*, *S*. *typhimurium*, and *E*. *coli* (Table 10). The mechanisms of fennel and its major components need further studies.

## 12. Coriander

Coriander (*Coriandrum sativum*), belonging to family Umbelliferae, is a native plant of the Mediterranean region and is widely cultivated in India, Russia, Central Europe, Asia, and Morocco. Coriander was widely applied in producing chutneys and sauces, flavoring pastry, cookies, buns, and tobacco products, and extensively employed for preparation of curry powder, pickling spices, sausages, seasonings, and food preservatives [4,118].

### 12.1. Antimicrobial Activities of Coriander

Duarte et al. [124] investigated the antimicrobial activities of coriander EO and its major compound, linalool, against *C. jejuni* and *C. coli* strains by the disc diffusion test, vapor-phase method and microdilution method. The MICs of coriander EO and linalool against *C. jejuni* and *C. coli* strains ranged between 0.5 and 1 μL/mL. Coriander EO also showed inhibitory effects on the biofilim formation of *Campylobacter* spp. Also, the antimicrobial activities of coriander EO against multidrug resistant pathogen, *Acinetobacter baumannii* (*A. baumannii*), were tested [125]. The MICs and MBCs were determined by a microdilution broth susceptibility assay. The MICs and MBCs of coriander EO against *A. baumannii* strains both ranged between 1 and 4 μL/mL. Another study investigated the synergistic antibacterial effects of coriander EO and six antibacterial drugs (cefoperazone, chloramphenicol, ciprofloxacin, gentamicin, tetracycline, and piperacillin) against two *A. baumannii* strains [126]. The results indicated that coriander EO showed synergistic action with chloramphenicol, ciprofloxacin, and tetracycline, and contributed to resensitizing *A. baumannii* to the action of chloramphenicol. Freires et al. [127] investigated the antifungal activities of EO from coriander leaves against *Candida* spp. The results showed that the MICs ranged from 15.6 to 31.2 µg/mL, and MFCs ranged from 31.2 to 62.5 µg/mL against *Candida* spp. for coriander EO. Sliva et al. [128] assessed the bacterial activities of coriander EO against 12 bacterial strains by microdilution broth susceptibility assay. The results indicated that coriander EO showed antimicrobial activities against all tested bacteria and showed bactericidal activities against bacteria except *B. cereus* and *E. faecalis*. The MICs of coriander against all tested bacteria ranged from 0.1% to 1.6% *v*/*v*, and MBCs ranged from 0.1% to 3.2% *v*/*v* except *B. cereus* and *E. faecalis.* Acimovic et al. [129] assessed the antifungal activities of EOs of six coriander accessions of different origins against *Colletotrichum acutatum* and *Colletotrichum gloeosporioides* using the inverted petriplate method. The results indicated that coriander EOs could inhibit *Colletotrichum* genus at higher application rates (≥0.16 μL/mL of air).

Singh et al. [130] reported the antifungal effects of coriander EO and oleoresin on eight fungi by the inverted petriplate and food poison techniques. The results of the former method showed that EO was highly active against *Curpularia palliscens*, *F. oxysporum*, *Fusarium monitiforme*, and *Aspergillus terreus* (*A. terreus*), and the oleoresin inhibited more than 50% mycelial zones for *F. oxysporum*, *A. niger*, and *A. terreus*. The results of the latter method indicated that EO 100% inhibited the growth of *A*. *terreus*, *A*. *niger*, *F*. *graminearum*, and *F*. *oxysporum*, but the oleoresin exhibited weaker fungitoxic activities, which only 100% inhibited the growth of *F*. *oxysporum*. In another study, the antimicrobial activities of ethanol and aqueous-ethanol extracts of coriander were investigated against *B*. *subtilis*, *S. aureus*, *P. vulgaris*, *E*. *coil*, *P. aeruginosa*, *K. peunomonia*, *L*. *monocytogenes*, and *C*. *albicans* [131]. Ethanol extract revealed the elevated antimicrobial activities against *P. vulgaris* and *C*. *albicans*, and was more potent against tested microorganisms. Besides, aqueous-ethanol extract exhibited the highest activities against *B*. *subtilis* and *L*. *monocytogenes*. Furthermore, the effect of microwaves on EO of coriander on its antimicrobial activities was also tested [83]. The antimicrobial effects against microorganisms of both microwave and conventionally roasted oils were similar and more effective than those of raw oils.

Coriander EO permeated the cell membranes, resulting in the loss of all cellular functions [4]. The mechanisms of antibacterial action of coriander EO on Gram-positive and Gram-negative bacteria are different and need further exploring. Coriander EO was found to bind to membrane ergosterol and increase ionic permeability, ultimately causing cell death of *C. albicans* [127].

### 12.2. Comparison of Antimicrobial Activities of Coriander and Other Spices

The antimicrobial activities of four spice EOs against isolated clinical specimens were compared using the diffusion method [87], and the results showed that coriander oil was active only against *Salmonella* sp. Dimic et al. [132] tested the antifungal activities of lemon EO, coriander extract and cinnamon extract against five molds (*A. parasiticus*, *Cladosporium cladosporioides* (*C. cladosporioides*), *Eurotium herbariorum*, *Penicillium chrysogenum*, and *Aspergillus carbonarius*) by the agar dilution method and vapor phase method. The results indicated that coriander extract had the best antifungal activities in the vapor phase as it completely inhibited *A. parasiticus*, *C. cladosporioides*, *E. herbariorum*, and *P. chrysogenum* at 4.17 μL/mL.

The papers cited are of high quality and indicated that coriander possessed significant antimicrobial activities at low concentrations against several pathogens such as *A. baumannii*, *Campylobacter* spp. at low MICs, MBCs, and MFCs (Table 11).

## 13. Galangal

Galangal (*Alpinia galangal*) (Table 12) has been used as a food additive in Thailand and other Asian countries since ancient time [133]. In a study, the antimicrobial activities of extracts of seven spices and herbs against *E*. *coli*, *S. typhimurium*, *L*. *monocytogenes*, and *S. aureus* were compared by the agar disc diffusion and broth dilution assays [93]. The hexane and ethanol extracts of galangal had strong antimicrobial activities against *S. aureus* (MIC < 0.625 mg/mL) and *L*. *monocytogenes* (MIC < 0.625 mg/mL at 24 h and 1.25 mg/mL at 48 h). Moreover, the synergistic antimicrobial effects of extract combination (galangal, rosemary, and lemon iron bark) on *S. aureus*, *L. monocytogenes*, *E*. *coli*, *S*. *typhimurium*, and *Clostridium perfringens* were evaluated [134]. Galangal and rosemary showed synergistic activities against *S. aureus* and *L*. *monocytogenes*, while galangal and lemon iron bark showed synergistic activities against *E*. *coli* and *S*. *typhimurium*. Additionally, Rao et al. [133] tested the antibacterial activities of galangal methanol, acetone, and diethyl ether extracts against *B*. *subtilis*, *E. aerogenes*, *E. cloacae*, *E. faecalis*, *E*. *coli*, *K. pneumoniae*, *P*. *aeruginosa*, *S*. *typhimurium*, *S. aureus*, and *S. epidermis* using agar well diffusion method and macrodilution method. Among the three solvents used, the activities of methanol extract at pH 5.5 were excellent against all the pathogens (MIC: 0.04–1.28 mg/mL, MBCs: 0.08–2.56 mg/mL). Another study also evaluated the antimicrobial activities of methanol extracts of four *Alpinia* strains against six strains of bacteria and four strains of fungi, using the disc diffusion assay [135]. The results demonstrated that galangal flower possessed the best effects on *M. luteus* and only the extract from galangal rhizome showed antifungal activity toward *A. niger*. The mechanisms of action of galangal have been rarely explored up till now.

## 14. Black Pepper

Black pepper (*Piper nigrum*) (Table 13) is largely used as a flavoring agent in foods. The antifungal effects of EO and acetone extract of black pepper on various pathogenic fungi were tested by the inverted petriplate technique and food poisoning technique [136]. The results showed that the EO was 100% controlled the mycelial growth of *F. graminearum*, while the acetone extract 100% inhibited mycelial growth of *Penicillium viridcatum* and *A. ochraceus*. In another study, the bacterial effects of EOs and acetone extracts of four spices on *S. aureus*, *B*. *cereus*, *B*. *subtilis*, *E*. *coli*, *S*. *typhi*, and *P*. *aeruginosa* were studied using the disk diffusion and poison food assay [137]. The results showed that black pepper extracts completely reduced colonies of *S. aureus*, *B*. *cereus*, and *B*. *subtilis* at 5 and 10 μL levels using the poison food method. Zarai et al. [138] evaluated the antimicrobial effects of various solvent extracts, piperine, and piperic acid from pepper against *E*. *coli*, *K. pneumonia*, *S*. *enterica*, *S. aureus*, *S*. *epidermidis*, *E*. *faecalis*, and *B*. *subtilis* by the agar diffusion assay and micro-well dilution assay. The results showed that the ethanol extract was the most effective to the tested bacteria with the MICs ranging from 156.25 μg/mL (*S. aureus* and *B*. *subtilus*) to 1250 μg/mL (*E*. *coli* and *K*. *pneumonia*).

Black pepper EO could cause physical and morphological alterations in the cell walls and membranes of *E. coli*, and then result in the leakage of electrolytes, ATP, proteins, and DNA materials [139]. Chemical components of black pepper and its mechanisms of antimicrobial action need further exploration.

## 15. Other Spices

The antimicrobial activities of the spices mentioned above against several common microorganisms are summarized in Table 14. Other spices—such as *Allium roseum* L., *Cinnamomum verum*, *Laurus nobilis*, *Myristica fragrans*, and *Pimpinella anisum*—were also proved to possess significant antifungal and antibacterial activities (Table 15).

## 16. Conclusions

The antibacterial and antifungal activities of commonly used spices have been summarized. Several spices—such as clove, oregano, thyme, cinnamon, and cumin—have exhibited significant antimicrobial activities against food spoilage bacteria like *B. subtilis* and *P. fluorescens*; pathogens like *S. aureus*, *V*. *parahaemolyticus*, and *S. typhimurium*; harmful fungi like *A. flavus* and *A*. *niger*; and even antibiotic resistant microorganisms such as MRSA. Therefore, these spices could be used to decrease the possibility of food poisoning and spoilage, to increase the food safety and shelf-life of products, and to treat some infectious diseases. In the future, as the combinations of several spices were proven to possess higher inhibitory effects on specific bacteria than those of individual spices, the interactions of more spices should be studied and evaluated to inhibit different microorganisms in different food products. Additionally, spices could be used in food packaging as published, but more studies are required to take the other aspects into consideration, such as how to prevent odor/flavor transferring from packages containing natural spice extracts to the packaged foods. Furthermore, spice products may be considered as an alternative to common antibiotics to treat infectious diseases. As the majority of the studies focused on the in vitro activities of spices against human pathogenic bacteria, in vivo studies and clinical trials are needed to be conducted in future. The mechanisms of antimicrobial action of spices remain to be clarified in order to make the best use of spices. Furthermore, the potential toxicity of spices on humans should be evaluated.

## Figures and Tables

**Table 1:** Antibacterial and antifungal activities of clove.

Type of Samples	Bacteria and Fungi	Main Results	Reference
Clove and cinnamon water extracts	*Staphylococcus aureus* and *Escherichia coli*	Both in vivo and in vitro results confirmed the efficacy of clove extract as natural antimicrobials.	[18]
Ultra-fine powders of ball-milled clove	*E*. *coli*, *S*. *aureus*, *Brochothrix thermosphacta*, *Lactobacillus rhamnosus*, *Pseudomonas fluorescens*	Clove powder showed a strong inhibitory effect with the minimum inhibitory concentrations (MICs) ranging from 1.0% to 2.0% *w*/*v*.	[19]
Cardamom, cinnamon, clove essential oils (EOs) and phenol	13 bacterial strains, 7 molds and 2 yeasts	Clove EO possessed the highest antimicrobial activities.	[21]
4 spice EOs	*Candida albicans*	Clove EO possessed the strongest activities against all *C*. *albicans* strains.	[22]
4 spice EOs	*Salmonella typhimurium*, *E*. *coli*, *Bacillus cereus*, *Listeria innocua*	Clove EO showed the most effective inhibition with the MICs ranging from 1.25% to 2.50% *v*/*v*.	[23]
9 spice EOs	*Morganella morganii*	Clove EO was the most effective with MIC of 0.13% *v*/*v*.	[17]
Ethanol extracts from 5 spices and herbs	*Listeria monocytogenes*, *S*. *aureus*, *Salmonella enterica*	Clove extract was the most effective against bacteria tested.	[24]
13 plant ethanol extracts	*L*. *monocytogenes*	Clove extract was the most effective with the MIC of 0.24 mg/mL.	[25]
90 plant extracts	*Clostridium* spp.	Clove water extract was the most effective among all the water extracts with the MIC ranging from 0.1% to 0.2%.	[26]
Ethyl acetate, acetone and methanol extracts of 12 plant species	*Kluyveromyces marxianus*, *Rhodotorula rubra*, *Klebsiella pneumoniae*, *Bacillus megaterium*, *Pseudomonas aeruginosa*, *S*. *aureus*, *E*. *coli*, *Enterobacter cloacae*, *Corynebacterium xerosis*, *Streptococcus faecalis*	Clove possessed the most effective inhibitory effects.	[27]
7 spices, their extracts and EOs	Microorganisms in apple cider	Clove products had the strongest antimicrobial activities compared with other spices tested.	[28]
Many spice and herb powders	*S*. *aureus*, *S. typhimurium*, *E*. *coli*, *B*. *cereus*	2% level of clove powder was more effective against *S*. *aureus* followed by *E*.*coli* and *S*. *typhimurium* under refrigeration.	[29]
EOs and functional extracts of cumin and clove.	*E*. *coli*, *S*. *typhimurium*, *S*. *aureus*, *B*. *cereus*, *L*. *monocytogenes*	The films of low molecular weight chitosan with a concentration of 2% of EO of clove and clove ethyl heptanoate extract had antimicrobial activities against most strains tested.	[30]
EOs and functional extracts of cumin, clove, and elecampane	*E*. *coli*, *S. typhimurium*, *B*. *cereus*, *S*. *aureus*, *L*. *monocytogenes*	Chitosan films added with clove EO showed the best inhibitory effects with the MICs of 500 mg/L.	[31]
3 spice EOs	*Enterobacteriaceae*, *S*. *aureus*, *Pseudomonas* sp., Lactic acid bacteria, *Brocithrix thermosphacta*	Clove EO was the most effective against microorganisms tested.	[32]

**Table 2:** Antibacterial and antifungal activities of oregano.

Type of Study	Bacteria and Fungi	Main Results	Reference
Oregano extract	*Salmonella gallinarum*, *Salmonella enteritidis*, *S*. *typhimurium*	Oregano extract had antibacterial effects on *Salmonella* serotypes.	[34]
EO-rich fractions of oregano	*S. aureus*, *B*. *subtilis*, *E*. *coli*, *P*. *aeruginosa*, *C*. *albicans*, *Aspergillus niger*	All of the supercritical fluid extraction fractions showed antimicrobial activities against all tested microorganisms.	[35]
Oregano EO	*C*. *albicans*, *Candida krusei*, *Candida tropicalis*, *B*. *cereus*, *E*. *coli*, *S. aureus*, *Yersinia enterocolitica*, *S*. *enterica*, *Serratia marcescens*	Heating treatment showed no significant effects on the antimicrobial activities of EO.	[36]
4 spice decoctions	*F*. *oxysporum* f. sp. *phaseoli*, *Macrophomina phaseoli*, *Botrytis cinerea*, *Rhizoctonia solani*, *Alternaria solani*, *Alternaria parasiticus*	The 10% level of oregano decoction was 100% inhibitive to mycelial growth in the culture medium.	[39]
4 plant aqueous extracts	*E*. *coli* and *B*. *subtilis*	Oregano extract had the highest antibacterial activities against all tested bacteria.	[40]
Oregano and marjoram EOs	*S. aureus* isolated from poultry meat.	All the isolates tested were sensitive to EO of oregano.	[41]
3 spice EOs	13 bacterial strains and 6 fungi	Oregano EO showed the most effective antibacterial activities.	[42]
6 spice EOs	*Staphylococcus xylosus*, *Staphylococcus carnosus*, *Lactobacillus sakei*, *Lactobacillus curvatus*, *Enterobacter gergoviae*, *Enterobacter amnigenus*	Oregano EO was the most effective.	[43]
8 spice EOs	*E*. *coli* strains isolated from poultry and cattle faeces.	Oregano EO was the most effective against *E. coli*.	[44]
Oregano and *A*. *sieberi* EOs	*Candida glabrata* isolated from patients with vulvovaginal candidiasis.	The MICs of oregano EO ranged from 0.5 to 1100 μg/mL for all tested *C*. *glabrata* isolates.	[45]
7 spice EOs and the majority constituents	33 *Staphylococcus* spp. isolates	Oregano and thyme EOs possessed the equal and strongest antimicrobial activities among EOs.	[46]
4 spice EOs	*S. aureus* and *E*. *coli* isolated from vongole and bacteria standard ATCC: *E*. *coli*, *S. aureus*, *Salmonella choleraesuis*	Oregano presented antimicrobial activities against all tested bacteria.	[47]
Various spice EOs	Microorganisms in fresh leaf lettuce and radish sprouts.	Oregano-2 (in the USA) oil was the most effective at maintaining the reduced levels of total mesophilic microorganisms.	[48]
Pure EOs of 4 spices and chitosan-EOs films	*L*. *monocytogenes* and *E*. *coli*	Both oregano EO alone and incorporated in the films possessed the best antimicrobial activities.	[49]

**Table 3:** Antibacterial and antifungal activities of thyme.

Type of Samples	Tested Bacteria and Fungi	Main Results	Reference
Thyme EO	*B*. *subtilis*, *S. aureus*, *Staphylococcus epidermidis*, *P*. *aeruginosa*, *E*. *coli*, *Mycobacterium smegmatis*, *C*. *albicans*, *Candida vaginalis*	MICs ranged from 75 to 1100 μg/mL for bacteria, and from 80 to 97 μg/mL for fungi.	[51]
Thyme EOs of 4 ontogenetic stages	*E*. *coli*, *Proteus mirabilis*, *Proteus vulgaris*, *S*. *typhimurium*, *S*. *marcescens*, *Y*. *enterocolitica*, *P*. *fluorescens*, *Pseudomonas putida*, *Micrococcus* spp., *S*. *flava*, *S. aureus*, *Bacillus licheniformis*, *Bacillus thuringiensis*, *L*. *innocua*	All the thyme EOs had significant antibacterial activities against the microorganisms tested.	[52]
4 *Thymus* species EOs	*P*. *aeruginosa*, *Cronobacter sakazakii*, *L*. *innocua*, *Streptococcus pyogenes*, *C*.*albicans*, *Saccharomyces cerevisiae*	Thyme EO was the most efficient on all the tested bacteria and yeast both in original and half-diluted concentrations.	[53]
5 spice hydrosols	*B*. *subtilis* and *S*. *enteritidis*	Thyme hydrosol was more effective than sage, peppermint, and black pepper.	[55]
6 plant hydrosols	*S. aureus*, *E*. *coli*, *S*. *typhimurium*, *P*. *aeruginosa*, *C*. *albicans*	15% hydrosol concentration of thyme completely inhibited *E*. *coli* and *S*. *typhimurium*.	[56]
5 plant EOs	*P*. *fluorescens*, *P*. *putida*, *Pseudomonas fragi*, *Brochothrix thermosphacta C*. *albicans*, *P*. *aeruginosa*	Thyme EO showed the highest antimicrobial activities with MICs ranging from 0.05% to 0.8% *w*/*v*.	[57]
5 aromatic plant EOs	13 *Vibrio alginolyticus* strains	The MICs of thyme EO ranged from 0.078 to 0.31 mg/mL, and MBCs ranged from 0.31 to 1.25 mg/mL.	[58]
5 spice EOs	*L*. *innocua*, *S*. *marcescens*, *P*. *fluorescens*	Only the thyme EO showed inhibition effects on all tested bacteria at all added doses.	[59]
4 spice EOs	*B*. *cereus*, *L*. *monocytogenes*, *S*. *typhimurium*, *E*. *coli*	MICs of thyme EO ranged from 0.312 to 1.25 μL/mL.	[60]
Various EOs	18 pathogens and 15 spoilage bacteria	Thyme EO showed the strongest antibacterial activities against spoilage bacteria.	[48]
17 spices and herbs	*Shigella sonnei* and *Shigella flexneri*	MICs of thyme ranged from 0.5% to 1% (*w*/*v*) depending on the *Shigella* strains used.	[61]
Thyme infusion, decoction and hydroalcoholic extracts	*S. aureus*, *S*. *epidermidis*, *E*. *coli*, *Klebsiella* spp., *P*. *aeruginosa*, *Enterobacter aerogenes*, *P*. *vulgaris*, *Enterobacter sakazakii*	Decoction presented the most pronounced effects.	[62]
Thyme EO, hydrosol and propolis extracts	Natural mycobiota on the surface of sucuk	Thyme EO and propolis extract provided reductions of 2.45 and 2.05 log CFU/g in yeast-mold counts respectively.	[63]
Polyphenolic fractions and oil fractions from 4 spice oilseeds	35 bacterial strains	Thyme oil fraction had the highest antibacterial activities comparing with other spices oilseeds.	[64]
Thymol, nisin, carvacrol, cymene	*Candida lusitaniae*	Thymol completely inhibited the yeast growth at concentrations over 1 mmol/L for at least 21 days at 25 °C.	[65]

**Table 4:** Antibacterial and antifungal activities of cinnamon.

Type of Samples	Tested Bacteria and Fungi	Mian Results	Reference
Cinnamon extract and oil	7 Gram-positive bacteria, 3 Gram-negative bacteria, and 7 fungi	Cinnamon oil was more effective than cinnamon extract with MICs ranging from 1.25% to 5% *v*/*v*.	[70]
Cinnamon, sodium benzoate, potassium sorbate	*E*. *coli*	*E*. *coli* was reduced by 1.6 log CFU/mL at 8 °C and 2.0 log CFU/mL at 25 °C by 0.3% cinnamon.	[71]
Cinnamon oil and liposome-encapsulated cinnamon oil	Methicillin resistant *Staphylococcus aureus* (MRSA)	Cinnamon oil possessed effective antibacterial activity and prominent anti-biofilm activity against MRSA.	[72]
4 spice aqueous extracts	*A*. *niger*, *Fusarium sambucinum*, *Pythium sulcatum*, *Rhizopus stolonifer*	0.05 g/mL of cinnamon extract completely inhibited *A*. *niger* and *P*. *sulcatum*, 0.10 g/mL of cinnamon extract completely inhibited *F*. *sambucinum*.	[74]
5 plant aqueous extracts	*S. aureus*, *Lactobacillus* sp., *B*. *thermosphacta*, *Pseudomonas* spp., *E*. *coli*	Cinnamon aqueous extract inhibited all the tested microorganisms at the concentration of 1%.	[75]
6 spice hydrosols	*S. aureus*, *E*. *coli*, *S. typhimurium*, *P. aeruginosa*, *C*. *albicans*	The percent inhibition ranged from 10% to 33.8% at 5% hydrosol of cinnamon.	[56]
6 spice diethyl ether extracts	*S. aureus*, *K*. *pneumoniae*, *P*. *aeruginosa*, *E*. *coli*, *Enterococcus faecalis*, *M*. *smegmatis*, *Micrococcus luteus*, *C*. *albicans*	Cinnamon possessed inhibitory activities against all the tested microorganisms.	[76]
Ethyl acetate, acetone, and methanol extracts from 12 plants	*K. pneumonia*, *B. megaterium*, *P. aeruginosa*, *S. aureus*, *E*. *coli*, *E. cloacae*, *C. xerosis*, *S. faecalis*, *K. marxianus*, *R. rubra*	The methanol extract of cinnamon showed antibacterial activities against all the microorganisms tested.	[27]
7 Indian spice ethanol extracts	215 enterococcal strains	Crude ethanol extract of cinnamon was the most effective against all the clinical isolates.	[77]
8 spice EOs	*B*. *cereus*, *E*. *coli*, *L*. *monocytogenes*, *S*. *rissen*, *P*. *fluorescens*, *S. aureus*, *Candida lipolytica*, *Hanseniaspora uvarum*, *Pichia membranaefaciens*, *Rhodotorula glutinis*, *Schizosaccharomyces pombe*, *Zygosaccharomyces rouxii*, *A. flavus*, *Aspergillus versicolor*, *A. parasiticus*, *Fusarium moniliforme*	Cinnamon EO possessed the strongest inhibition effects with the MICs ranging from 0.015 to 2.0 mg/mL.	[78]
10 spice EOs	*B*. *cereus*, *B*. *subtilis*, *E*. *coli*, *K. pneumoniae*, *L*. *monocytogenes*, *P*. *aeruginosa*, *S. aureus*, *S. enterica*, *S. marcencens*, *Y. enterocolitica*	Cinnamon EO was efficient in inhibiting all tested bacterial strains.	[79]

**Table 5:** Antibacterial and antifungal activities of cumin.

Type of Study	Bacteria and Fungi	Main Results	Reference
Cumin EO	*E*. *coli*, *S. aureus*, *S*. *faecalis*, *P*. *aeruginosa*, *K*. *pneumoniae*	*E*. *coli*, *S. aureus*, and *S*. *faecalis* were sensitive to various cumin EO dilutions.	[81]
Cumin seeds EO	1230 fungal isolates obtained from food samples	The EO was fungicidal against most of the fungal species at MIC of 0.6 μL/mL.	[82]
Cumin seeds EOs	*E*. *coli*, *S. aureus*, *P*. *aeruginosa*, *A*. *niger*, *A*. *parasiticus*, *C*. *albicans*	Both microwave and conventionally (oven) roasted cumin oils showed higher effects than raw oils.	[83]
Cumin EO	*E*. *coli*, *P*. *aeruginosa*, *B*. *cereus*, *S. aureus*	ã-Irradiation to cumin seeds at 10 and 25 kGy had no significant effects on the antibacterial effects.	[80]
Aqueous infusions and aqueous decoctions from kalonji, cumin and poppy seed	188 bacterial isolates isolated from oral cavity of apparently healthy individuals	Aqueous decoction of cumin inhibited 73% of the tested bacteria.	[84]
Cumin and *C*. *carvi* EOs	*E. coli*, the genera *Pseudomonas*, *Clavibacter*, *Curtobacterium*, *Rhodococcus*, *Erwinia*, *Xanthomonas*, *Ralstonia*, *Agrobacterium*	Cumin EO showed antibacterial activities against all tested bacteria except *Pseudomonas viridiflava*.	[85]
9 spice EOs	*S*. *typhimurium*, *B*. *cereus*, *S. aureus*, *E*. *faecalis*, *E*. *coli*. *Y*. *enterocolitica*, *S*. *cerevisiae*, *Candida rugosa*, *Rhizopus oryzae*, *A*. *niger*	Cumin EO was effective against all bacterial species and two fungi (*S*. *cerevisiae* and *Candida rugosa*).	[86]
4 spice EOs	*S. aureus*, *Salmonella* sp., *E*. *coli*, *P*. *aeruginosa*, etc.	Cumin EO was effective against *E*. *coli*, *P*. *aeruginosa* and *Salmonella* sp.	[87]
6 spice EOs	*L*. *curvatus*, *L*. *sakei*, *S*. *carnosus*, *S*. *xylosus*, *E*. *gergoviae*, *E*. *amnigenus*	Cumin EO was the second effective among tested spices.	[43]
5 spice EOs	*M. luteus*, *B*. *megaterium*, *Brevibacillus brevis*, *E. faecalis*, *Pseudomonas pyocyaneus*, *M. smegmatis*, *E*. *coli*, *Aeromonas hydrophila*, *Y. enterocolitica*, *S. aureus*, *S. faecalis*, *S. cerevisiae*, *Kluvyeromyces fragilis*	Cumin inhibited all tested bacteria and fungi and showed synergistic and antagonistic effect with antibiotics.	[88]
EOs of 9 spices in combination	*B*. *cereus*, *L*. *monocytogenes*, *M*. *luteus*, *S. aureus*, *E*. *coli*, *S*. *typhimurium*	Coriander/cumin seed oil combination showed synergistic interactions on antibacterial activities.	[89]

**Table 6:** Antibacterial and antifungal activities of rosemary.

Type of Study	Bacteria and Fungi	Main Results	Reference
Rosemary EO	*Leuconostoc mesenteroides*, *Lactobacillus delbruekii*, *S*. *cerevisiae*, *C*. *krusei*	Rosemary EO showed higher effects against bacteria tested than yeasts.	[91]
Rosemary and sage EOs	*C*. *albicans*, *Trichophyton mentagrophytes*, *Trichophyton tonsurans*, *Trichophyton rubrum*, *Epidermophyton floccosum*, *Microsporum canis*, *P*. *aeruginosa*, *E*. *coli*, *S*. *typhimurium*, *S*. *enteritidis*, *Shigella sonei*, *Micrococcus flavus*, *Sarcina lutea*, *S. aureus*, *S*. *epidermidis*, *B*. *subtilis*	The EO of rosemary showed significant antifungal activities and antibacterial activities.	[92]
7 spice and herb extracts	*E*. *coli*, *S*. *typhimurium*, *L*. *monocytogenes*, *S. aureus*	The hexane extract of rosemary exhibited significantly higher antibacterial activities than ethanol and water extracts.	[93]
5 plant ethanol extracts	*B*. *cereus*, *E*. *coli*, *P*. *aeruginosa*, *S. aureus*, *L*. *monocytogenes*	Rosemary extract was the most effective against all the tested microorganisms.	[94]
14 spice ethanol extracts and their mixture	*L*. *monocytogenes*, *E*. *coli*, *P*. *fluorescens*, *L*. *sake*	The mixture of rosemary and liquorice extracts was the most effective against all tested bacteria.	[95]
5 spice aqueous extracts	8 Gram-positive bacteria and 12 Gram-negative bacteria	MICs ranged from 0.125 to 0.5 mg/mL for Gram-positive bacteria and 0.25–0.5 mg/mL for Gram-negative bacteria.	[96]
2 spice and herb extract combinations	*L*. *monocytogenes*, *S. aureus* and naturally present spoilage microflora on cooked ready-to-eat shrimp stored for 16 days at 4 or 8 °C	Both combination of galangal, rosemary, and lemon and combination of galangal and rosemary significantly reduced levels of aerobic bacteria and lactic acid bacteria.	[97]
Rosemary, mint and a mixture of tocopherols	Microorganisms from the sausages	The addition of rosemary resulted in significant inhibition of microbial growth and showed possible synergistic effects with mint.	[90]
5 spice EOs	*M. luteus*, *B. megaterium*, *B*. *brevis*, *E*. *faecalis*, *P*. *pyocyaneus*, *M. smegmatis*, *E*. *coli*, *A. hydrophila*, *Y. enterocolitica*, *S. aureus*, *S*. *faecalis*, *S. cerevisiae*, *K. fragilis*	Rosemary EO showed synergic effects with cephalothin.	[88]

**Table 7:** Antibacterial and antifungal activities of garlic.

Type of Study	Bacteria and Fungi	Main Results	Reference
Fresh garlic, garlic powder, garlic oil	Microorganisms in raw chicken sausage	The order of antimicrobial activities were fresh garlic > garlic powder > garlic oil > butylated hydroxyanisole.	[100]
Garlic powder	*S. aureus*, *E*. *coli*, *S*. *typhimurium*, *B*. *cereus*, and a mixed lactic culture consisting of *Lactobacillus delbrueckii* subsp. bulgaricus and *Streptococcus thermophilus*	Fresh garlic produced the greatest inhibition followed by freeze-dried powder.	[99]
Chopped garlic	Microorganisms in ground beef and raw meatball	Chopped garlic had slowing-down effects on microbiological growth.	[101]
5 spice extracts	20 serogroups of *E*. *coli*, 8 serotypes of *Salmonella*, *L*. *monocytogenes* and *A*. *hydrophila*	Garlic extract exhibited significant activities against microorganisms except *L. monocytogenes* at all concentrations.	[103]
3 ethanol extracts	*K*. *pneumoniae*, *S. aureus*, *M*. *morganii*, *C*. *albicans*, *E*. *coli*, *P*. *vulgaris*	Garlic extract exerted superior antibacterial activities at all concentrations	[104]
6 spice crude ethanol, hexane and acetone extracts	*E*. *coli*, *S. aureus*, *S*. *flexneri*, *Streptococcus pneumoniae*	Garlic was the most effective against all the tested pathogens except *S*. *flexneri*.	[105]
7 spice crude extracts	*Phoma exigua*, *Fusarium nygamai*, *R*. *solani*	Cold water extract of garlic exhibited good antifungal activities against all three tested fungi.	[106]
Garlic aqueous extract	*S. aureus*	The first and second week samples were significantly decreased by all the 1, 2, and 3-mL garlic extracts.	[98]
5 spice hydrosols	*B*. *subtilis* and *S*. *enteritidis*	Garlic hydrosols demonstrated stronger antibacterial activities than other spices hydrosols.	[54]

**Table 8:** Antibacterial and antifungal activities of ginger.

Type of Sample	Bacteria and Fungi	Main Results	Reference
3 spice extracts and EOs	5 strains of *L*. *monocytogenes*, 4 strains of *S*. *typhimurium* DT104	Commercial EOs of ginger inhibited all *L*. *monocytogenes* at ≤ 0.6	[111]
Ginger EO and oleoresin	*Aspergillus terrus*, *A. niger*, *A. flavus*, *Trichothecium roseum*, *Fusarium graminearum*, *Fusarium oxysporum*, *Fusarium monoliforme*, *Curvularia palliscens*	EO and oleoresin of ginger were 100% antifungal against *F*. *oxysporum* and *A*. *niger*, respectively.	[109]
Ginger ethanol and *n*-hexane extracts	*Porphyromonas gingivalis*, *Porphyromonas endodontalis*, *Prevotella intermedia*	Only [10]-gingerol and [12]-gingerol effectively inhibited the growth of tested bacteria at a MIC range of 6–30 µg/mL.	[107]
Ginger oil extracted by hydrodistillation and solvent extraction method	*B*. *subtilis*, *Bacillus nutto*, *P*. *aerugenosa*, *Rhodoturola* sp., *Samonella newport*, *S*. *enteritidis*, *Fusarium* sp.	Extracts obtained by both extraction methods inhibited listed microorganisms.	[108]
Ginger and mustard EOs	*Vibrio* species	Ginger and mustard EOs inhibited the growth of *Vibrio parahaemolyticus* and *Vibrio vulnificus*.	[110]
5 spice extracts	20 serogroups of *E*. *coli*, 8 serotypes of *Salmonella*, *L*. *monocytogenes* and *A*. *hydrophila*.	Ginger extract possessed inhibitory effects on two serogroups of *E. coli*.	[102]
4 spice water extracts	*A*. *niger*, *F*. *sambucinum*, *P*. *sulcatum*, *R*. *stolonifera*	Ginger extract significantly inhibited the mycelial growth of tested microorganisms.	[74]
7 spice crude extracts	*P*. *exigua*, *F*. *nygamai*, *R*. *solani*	In the case of the hot water extracts, garlic and ginger showed the best antifungal activities.	[106]
7 spice ethanol extracts	215 enterococcal strains isolated from clinical samples	Ginger was found to have antibacterial activities against all the isolates.	[76]

**Table 9:** Antibacterial and antifungal activities of basil.

Type of Samples	Bacteria and Fungi	Main Results	Reference
EO from aerial parts of basil	*S. aureus*, *E*. *coli*, *B*. *subtilis*, *Pasteurella multocida*, *A*. *niger*, *Mucor mucedo*, *F*. *solani*, *Botryodiplodia theobromae*, *R*. *solani*	All the tested microorganisms were sensitive to EOs of basil.	[113]
Chloroform, acetone and methanol extracts of basil	*E. gallinarum*, *E. faecalis*, *B*. *subtilis*, *E*. *coli*, *Shigella* sp., *S. pyogenes*, *S. aureus*, *L*. *monocytogenes*, *P*. *aeruginosa*, *S*. *cerevisiae*, *C*. *albicans*, *C. crusei*	The methanol extract inhibited *P*. *aeruginosa*, *Shigella* sp., *L*. *monocytogenes*, *S. aureus*, and two strains of *E*. *coli*.	[114]
Basil extracts	*Fusarium oxysporum*, *Fusarium proliferatum*, *Fusarium subglutinans*, *Fusarium verticillioides*	At the concentration of 1.50% *v*/*v*, basil extract completely inhibited *Fusarium* spp. tested.	[115]
EOs from 12 cumin cultivars	*B*. *cereus*, *M*. *flavus*, *S. aureus*, *E*. *faecalis*, *E*. *coli*, *P*. *aeruginosa*, *S*. *typhimurium*, *L*. *monocytogenes*, *7 fungi*, *Aspergillus fumigatus*, *A*. *niger*, *A*. *versicolor*, *Aspergillus ochraceus*, *Penicillium funiculosum*, *Penicillium ochrochloron*, *Trichoderma viride*	MICs of basil EOs ranged from 0.009 to 23.48 μg/mL for bacteria and 0.08–5.00 μg/mL for fungi.	[116]
7 spice EOs	*A. flavus*	Basil EO completely inhibited *A*. *flavus* at 150 μL/100 mL.	[117]
8 spice EOs	Histamine-producing bacteria including *M*. *morganii*	Basil EO inhibited *M*. *morganii* with the MIC of 2.39 mg/mL.	[17]

**Table 10:** Antibacterial and antifungal activities of fennel.

Type of Sample	Bacteria and Fungi	Main Results	Reference
Fennel seeds EO	*Streptococcus mutans*	MICs: 80 ppm	[120]
Fennel seeds EO	*Streptomyces albus*, *B. subtilis*, *S. typhimurium*, *P*. *aeruginosa*, *Shigella dysenteriae*, *E. coli*	EO of fennel seeds inhibited several foodborne pathogens with lowest MIC of 0.125 mg/mL.	[119]
Fennel crude extract	*E*. *coli*, *S*. *blanc*, *P*. *merabilis*, *P*. *vulgaris*, *S*. *epidemidis*, *Staphylococcus saprophyticus*, *A*. *versicolor*, *A*. *fumigates*, *Penicilium camemberti*	Fennel crude extract had antimicrobial activities against all nine microorganisms, especially fungi.	[121]
Fennel EO and acetone extract	*A*. *niger*, *A*. *flavus* , *Aspergillus oryzae*, *A*. *ochraceus*, *F*. *graminearum*, *F*. *moniliforme*, *P*. *ctrium*, *Penicillium viridicatum*, *Penicillium madriti*, *Curvularia lunata*	Fennel EO showed complete zone inhibition against several strains at 6 μL dose.	[118]
Cumin and fennel EOs	*S*. *typhimurium* and *E*. *coli*	The MICs of fennel EO was 0.031% *v*/*v* against *S. typhimurium* and 0.062% *v*/*v E. coli*.	[122]
8 spice methanol and ethanol extracts	*B*. *subtilis*, *E*. *faecalis*, *L*. *innocua*, *E*. *coli*, *P*. *putida*, *Providencia stuartii*, *Acetobacter calcoaceticus*	Fennel seeds extracts showed the largest zones of inhibitions in six out of the seven bacteria.	[123]

**Table 11:** Antibacterial and antifungal activities of coriander.

Type of Sample	Bacteria and Fungi	Main Results	Reference
Coriander EO and linalool	*Campylobactor jejuni* and *Campylobactor coli* strains	MICs ranged between 0.5 and 1 mL/mL.	[124]
Coriander EO	*A. baumannii* strains	MICs and MBCs ranged between 1 and 4 μL/mL.	[125]
Coriander EO and 6 antibacterial drugs	*A. baumannii* strains	Coriander EO showed synergistic action with chloramphenicol, ciprofloxacin, and tetracycline.	[126]
Coriander leaves EO	*Candida* spp.	MICs ranged from 15.6 to 31.2 µg/mL, and MFCs ranged from 31.2 to 62.5 µg/mL.	[127]
Coriander EO	12 bacterial strians	MICs of coriander against all tsted bacteria ranged from 0.1% to 1.6%, *v*/*v*.	[128]
EOs of 6 coriander accessions	*Colletotrichum acutatum* and *Colletotrichum gloeosporioides*	Coriander EOs could inhibit *Colletotrichum* genus at higher application rates.	[129]
Coriander EO and oleoresin	*Aspergillus terreus*, *A*. *niger*, *F*. *graminearum*, *F*. *oxysporum*	Both EO and oleoresin of coriander were effective against tested fungi.	[130]
Coriander ethanol and aqueous-ethanol extracts	*B*. *subtilis*, *S. aureus*, *P*. *vulgaris*, *E*. *coil*, *P*. *aeruginosa*, *K*. *peunomonia*, *L*. *monocytogenes*, *C*. *albicans*	The ethanol extract showed clear difference and more potent against tested microorganisms in comparison with the aqueous-ethanol extract.	[131]
Coriander EO	-	Microwave and conventionally roasted oils exhibit similar antimicrobial effects but were higher effect than raw oils.	[83]
4 spice EOs	Microorganisms isolated from clinical specimens of patients	Coriander oil was active only against *Salmonella* sp.	[87]
Lemon EO, coriander extract and cinnamon extract	*A. parasiticus*, *Cladosporium cladosporioides*, *Eurotium herbariorum*, *Penicillium chrysogenum* and *Aspergillus carbonarius*	Coriander extract had the best antifungal activities in the vapor phase	[132]

**Table 12:** Antibacterial and antifungal activities of galangal.

Type of Sample	Bacteria and Fungi	Main Results	Reference
7 spice and herb extracts	*E*. *coli*, *S*. *typhimurium*, *L*. *monocytogenes*, *S. aureus*	Galangal hexane and ethanol extracts had strong antimicrobial activities against *S. aureus* and *L*. *monocytogenes*.	[93]
Combination of extracts from galangal, rosemary and lemon iron bark	*S. aureus*, *L*. *monocytogenes*, *E*. *coli*, *S*. *typhimurium*, *Clostridium perfringens*	Galangal showed synergistic activities against tested microorganisms with rosemary and lemon iron bark.	[134]
Galangal methanol, acetone and diethyl ether extracts	*B*. *subtilis*, *E*. *aerogenes*, *E*. *cloacae*, *E*. *faecalis*, *E*. *coli*, *K*. *pneumoniae*, *P*. *aeruginosa*, *S*. *typhimurium*, *S. aureus*, *S*. *epidermis*	The activities of methanol extract at pH 5.5 were excellent with MICs ranging from 0.04 to 1.28 mg/mL.	[133]
4 *Alpinia* strains methanol extracts	6 strains of bacteria and 4 strains of fungi	Galangal flower possessed the highest activity against *M*. *luteus* and only the extract from galangal rhizome showed antifungal activity toward *A*. *niger*.	[135]

**Table 13:** Antibacterial and antifungal activities of black pepper

Type of Samples	Bacteria and Fungi	Main Results	Reference
Black pepper EO and acetone extract	*A. flavus*, *A. ochraceus*, *A.oryzae*, *A. niger*, *F. moniliforme*, *F. graminearum*, *Penicillium citrinum*, *Penicillium viridcatum*, *P*. *madriti*, *Curvularia lunata*	The EO was effective against *F*. *graminearum*, while the acetone extract was effective against *P*. *viridcatum* and *A*. *ochraceus*.	[136]
4 spice EOs and acetone extracts	*S. aureus*, *B*. *cereus*, *B*. *subtilis*, *E*. *coli*, *S*. *typhimurium*, *P*. *aeruginosa*	Black pepper extracts showed complete reduction of colonies against tested bacterial strains at 5 and 10 μL levels.	[137]
Various solvent extracts, piperine and piperic acid from pepper	*E*. *coli*, *K*. *pneumonia*, *S*. *enterica*, *S. aureus*, *S*. *epidermidis*, *E*. *faecalis*, *B*. *subtilis*	The ethanol extract was the most effective with the MICs ranging from 156.25 to 1250 μg/mL.	[138]

**Table 14:** Antimicrobial activities of spices against several common microorganisms.

Bacteria and Fungi	Spices	Type of Samples	Reference
*E. coli*	Clove	Aqueous extract	[18]
		Acetone extract	[27]
		EO	[23,30,31]
		Ethyl acetate extract	[27]
		Ethyl heptanoate extract	[30]
		Methanol extract	[27]
		Powder	[19,29]
	Oregano	Aqueous extract	[40]
		EO	[36,44,47,49]
		EO-rich fractions	[35]
	Thyme	Decoction	[62]
		EO	[51,52,60]
		Hydroalcoholic extract	[62]
		Hydrosol	[56]
		Infusion	[62]
	Cinnamon	Acetone extract	[27]
		Aqueous extract	[75]
		Diethyl ether extract	[76]
		EO	[78,79]
		Ethyl acetate extract	[27]
		Hydrosol	[56]
		Methanol extract	[27]
		Powder	[71]
	Cumin	EO	[80,81,83,85,86,87,88,89]
	Rosemary	Aqueous extract	[93]
		EO	[88,92]
		Ethanol extract	[93,94,95]
		Hexane extract	[93]
	Garlic	Acetone extract	[105]
		Aqueous extract	[103]
		Ethanol extract	[104,105]
		Hexane extract	[105]
		Powder	[99]
	Ginger	Aqueous extract	[103]
	Basil	Acetone extract	[114]
		Chloroform extract	[114]
		EO	[113,116]
		Methanol extract	[114]
	Fennel	Crude extract	[121]
		EO	[121,122]
		Ethanol extract	[123]
		Methanol extract	[123]
	Coriander	Aqueous-ethanol extract	[131]
		Ethanol extract	[131]
	Galangal	Acetone extract	[133]
		Diethyl ether extract	[133]
		Hexane extract	[134]
		Methanol extract	[133]
	Black pepper	Acetone extract	[137]
		EO	[137]
		Ethanol extract	[138]
*S. aureus*	Clove	Acetone extract	[27]
		Aqueous extract	[18]
		EO	[30,31,32]
		Ethanol extract	[24]
		Ethyl heptanoate extract	[30,31]
		Methanol extract	[27]
		Powder	[19,29]
	Oregano	EO	[36,41,47]
		EO-rich fractions	[35]
	Thyme	Decoction	[62]
		EO	[51,52]
		Hydroalcoholic extract	[62]
		Infusion	[62]
	Cinnamon	Acetone extract	[27]
		Aqueous extract	[75]
		Diethyl ether extract	[76]
		EO	[72,78,79]
		Ethyl acetate extract	[27]
		Hydrosol	[56]
		Methanol extract	[27]
	Cumin	EO	[80,81,83,86,88,89]
	Rosemary	EO	[88,92]
		Ethanol extract	[94,97]
		Hexane extract	[93]
	Garlic	Aqueous extract	[98]
		Acetone extract	[105]
		Ethanol extract	[104,105]
		Hexane extract	[105]
		Powder	[99]
	Basil	Acetone extract	[114]
		Chloroform extract	[114]
		EO	[113,116]
		Methanol extract	[114]
	Coriander	Aqueous-ethanol extract	[131]
		Ethanol extract	[131]
	Galangal	Acetone extract	[133]
		Diethyl ether extract	[133]
		Ethanol extract	[93]
		Hexane extract	[93,134]
		Methanol	[133]
	Black pepper	Acetone extract	[137]
		EO	[137]
		Ethanol extract	[138]
*L. monocytogenes*	Clove	Ethanol extract	[24,25]
		Ethyl heptanoate extract	[30,31]
		EO	[30,31]
	Oregano	EO	[49]
	Thyme	EO	[60]
	Cinnamon	EO	[78,79]
	Cumin	EO	[89]
	Rosemary	Aqueous extract	[93]
		Ethanol extract	[93,94,95,97]
		Hexane extract	[93]
	Garlic	Aqueous extract	[103]
	Ginger	EO	[111]
	Basil	Acetone extract	[114]
		Chloroform	[114]
		EO	[116]
		Methanol extract	[114]
	Coriander	Aqueous-ethanol extract	[131]
		Ethanol extract	[131]
	Galangal	Ethanol extract	[93]
		Hexane extract	[93,134]
*S. typhimurium*	Clove	EO	[23,30,31]
		Ethyl heptanoate extract	[30]
		Powder	[29]
	Oregano	Extract	[34]
	Thyme	EO	[60,52]
		Hydrosol	[56]
	Cumin	EO	[86,89]
	Rosemary	Aqueous extract	[93]
		EO	[92]
		Ethanol extract	[93]
		Hexane extract	[93]
	Garlic	Powder	[99]
	Basil	EO	[116]
	Fennel	EO	[121,122]
	Galangal	Acetone extract	[133]
		Diethyl ether extract	[133]
		Hexane extract	[134]
		Methanol extract	[133]
	Black pepper	Acetone extract	[137]
		EO	[137]
*P*. *aeruginosa*	Clove	Acetone extract	[27]
		EO	[32]
		Ethyl acetate extract	[27]
		Methanol extract	[27]
	Oregano	EO-rich fractions	[35]
	Thyme	Decoction	[62]
		EO	[51,53,57]
		Hydroalcoholic extract	[62]
		Infusion	[62]
	Cinnamon	Diethyl ether extract	[76]
		EO	[79]
		Hydrosol	[56]
		Methanol extract	[27]
	Cumin	EO	[83,87]
	Rosemary	EO	[92]
		Ethanol extract	[94]
	Ginger	EO	[108]
	Basil	Acetone extract	[114]
		Chloroform	[114]
		EO	[116]
		Methanol extract	[114]
	Fennel	EO	[121]
	Coriander	Aqueous-ethanol extract	[131]
		Ethanol extract	[131]
	Galangal	Acetone extract	[133]
		Diethyl ether extract	[133]
		Methanol extract	[133]
	Black pepper	Acetone extract	[137]
		EO	[137]
*B*. *subtilis*	Oregano	Aqueous extract	[40]
		EO-rich fractions	[35]
	Thyme	EO	[51]
		Hydrosol	[55]
	Cinnamon	EO	[79]
	Rosemary	EO	[92]
	Garlic	Hydrosol	[55]
	Ginger	EO	[108]
	Basil	EO	[113]
	Fennel	EO	[121]
		Ethanol extract	[123]
		Methanol extract	[123]
	Coriander	Aqueous-ethanol extract	[131]
		Ethanol extract	[131]
	Galangal	Acetone extract	[133]
		Diethyl ether extract	[133]
		Methanol extract	[133]
	Black pepper	Acetone extract	[137]
		EO	[137]
		Ethanol extract	[138]
*B. cereus*	Clove	EO	[23,30,31]
		Ethyl heptanoate extract	[30]
	Oregano	EO	[36]
	Thyme	EO	[60]
	Cinnamon	EO	[78,79]
	Cumin	EO	[80,86,89]
	Rosemary	Ethanol extract	[94]
	Garlic	Powder	[99]
	Basil	EO	[116]
	Black pepper	Acetone extract	[137]
		EO	[137]
*E*. *faecalis*	Cinnamon	Diethyl ether extract	[76]
	Cumin	EO	[86,88]
	Rosemary	EO	[88]
	Basil	EO	[116]
	Fennel	Ethanol extract	[123]
		Methanol extract	[123]
	Galangal	Acetone extract	[133]
		Diethyl ether extract	[133]
		Methanol extract	[133]
	Black pepper	Ethanol extract	[138]
*E*. *faecalis*	Cinnamon	Diethyl ether extract	[76]
	Cumin	EO	[86,88]
	Basil	EO	[116]
	Fennel	Ethanol extract	[123]
		Methanol extract	[123]
	Galangal	Acetone extract	[133]
		Diethyl ether extract	[133]
		Methanol extract	[133]
	Black pepper	Ethanol extract	[138]
*K. pneumoniae*	Clove	Acetone extract	[27]
		Ethyl acetate	[27]
		Methanol extract	[27]
	Cinnamon	Acetone extract	[27]
		Diethyl ether extract	[76]
		EO	[79]
		Ethyl acetate	[27]
		Methanol extract	[27]
	Garlic	Ethanol extract	[104]
	Galangal	Acetone extract	[133]
		Diethyl ether extract	[133]
		Methanol extract	[133]
	Black pepper	Ethanol extract	[138]
*P*. *vulgaris*	Thyme	Decoction	[62]
		EO	[52]
		Hydroalcoholic extract	[62]
		Infusion	[62]
	Garlic	Ethanol extract	[104]
	Fennel	Crude extract	[121]
	Coriander	Aqueous-ethanol extract	[131]
		Ethanol extract	[131]
*P*. *fluorescens*	Clove	Powder	[19]
	Thyme	EO	[57,59,52]
	Cinnamon	EO	[78]
	Rosemary	Ethanol extract	[95]
*L*. *innocua*	Clove	EO	[23]
	Thyme	EO	[52,59,52]
	Fennel	Ethanol extract	[123]
		Methanol extract	[123]
*S*. *faecalis*	Clove	Acetone extract	[27]
		Ethyl acetate	[27]
		Methanol extract	[27]
	Cinnamon	Acetone extract	[27]
		Ethyl acetate	[27]
		Methanol extract	[27]
	Cumin	Decoctions	[81]
		EO	[88]
		Infusions	[81]
*S*. *enteritidis*	Thyme	Hydrosol	[55]
	Garlic	Hydrosol	[55]
	Ginger	EO	[108]
*M*. *luteus*	Cinnamon	Diethyl ether extract	[76]
	Cumin	EO	[88]
	Galangal	Methanol extract	[135]
*B. megaterium*	Clove	Acetone extract	[27]
		Ethyl acetate	[27]
		Methanol extract	[27]
	Cinnamon	Acetone extract	[27]
		Ethyl acetate	[27]
		Methanol extract	[27]
	Cumin	EO	[88]
*A. hydrophila*	Cumin	EO	[88]
	Garlic	Aqueous extract	[103]
*S*. *epidermidis*	Thyme	Decoction	[62]
		EO	[51]
		Hydroalcoholic extract	[62]
		Infusion	[62]
	Black pepper	Ethanol extract	[138]
*C. albicans*	Clove	EO	[22]
	Oregano	EO	[35,36]
	Thyme	EO	[51,57]
	Cinnamon	Hydrosol	[56,76]
		Diethyl ether extract	[76]
	Cumin	EO	[83]
	Rosemary	EO	[92]
	Garlic	Ethanol extract	[104]
	Coriander	Aqueous-ethanol extract	[131]
		EO	[127]
		Ethanol extract	[131]
*A*. *niger*	Oregano	EO-rich fraction	[35]
	Cinnamon	Aqueous extract	[74]
	Cumin	EO	[83]
	Ginger	Aqueous extract	[74,109]
		EO	[109]
		Oleoresin	[109]
	Basil	EO	[113,116]
	Fennel	Acetone extract	[118]
		EO	[118]
	Coriander	EO	[130]
	Galangal	Methanol extract	[135]
*A*. *flavus*	Basil	EO	[117]
	Fennel	Acetone extract	[118]
		EO	[118]
*F*. *oxysporum*	oregano	Decoction	[39]
	ginger	EO	[109]
	basil	Extract	[115]
	coriander	EO	[130]
		Oleoresin	[130]
*F*. *graminearum*	Fennel	Acetone extract	[118]
		EO	[118]
	Coriander	EO	[130]
		Oleoresin	[130]
	Black pepper	Acetone extract	[136]
		EO	[136]
*S*. *cerevisiae*	Thyme	EO	[52]
	Cumin	EO	[86]

**Table 15:** Antibacterial and antifungal activities of other spices.

Spices	Type of Samples	Bacteria and Fungi	Main Results	Reference
*Achillea* species	Ethanol extract	*K*. *pneumoniae*, *E*. *cloacae*, *S*. *typhimurium*, *S*. *epidermis*, *E*. *coli*, *E*. *aerogenes*, *S. aureus*, *Klebsiella oxytoca*, *S*. *pyogenes*, *P*. *aeruginosa*, *C*. *albicans*	*Achillea* species showed a broad spectrum of strong antibacterial activities against all tested microorganisms.	[140]
*Achillea millefolium*	Ethanol extract	*S. aureus*, *S*. *enteritidis*, *E*. *coli*, *S*. *pneumoniae*, *K*. *pneumoniae*, *P*. *aeruginosa*, *E*. *aerogenes*, *P. mirabilis*, *A*. *niger*, *C*. *albicans*	The antibacterial activities of *A*. *millefolium* were greater or similar to other penicillin derivatives but lesser than Ampicillin.	[141]
*Aframomum corrorima*	Seeds, pods, leaves and rhizomes extract	*A*. *flavus* and *Penicillum expansum*	*A. corrorima* crude seed extract was the most active against *A*. *flavus* and *P*. *expansum* at concentration of 0.4 mg/mL.	[142]
*Allium hirtifolium* Boiss.	Hydromethanol extract	MRSA, *S*. *epidermidis*, *S*. *pneumoniae*, *E*. *coli*, *S*. *typhimurium*, *P*. *mirabilis*, *K*. *pneumoniae*	*A*. *hirtifolium* extract was effective against 10 species of pathogenic bacteria with MICs ranging from 1.88 to 7.50 mg/mL.	[143]
*Allium roseum* L.	Extracts of bulbs, leaves, flowers and seeds by 3 extraction methods	*S*. *aureu*, *S*. *epidermidis*, *M. luteus*, *B*. *cereus*, *B*. *subtilis*, *E*. *faecalis*, *S*. *typhimurium*, *E*. *coli*, *P*. *aeruginosa*, *C*. *albicans*	*A*. *roseum* extract showed very significant antimicrobial activities to strains such as *C*. *albicans* (MICs: 1.00–3.44 μg/μL) and *E*. *coli* (MICs: 2.00–3.44 μg/μL).	[144]
*Allium ursinum* L.	Pressurized-liquid extract	*S. aureus* and *A*. *niger*	*A*. *ursinum* extract showed antimicrobial activities against *S. aureus* with DIZs of 12 and 10 mm (two parallel determinations) and *A*. *niger* of 6 mm.	[145]
*Amomum kravanh*	EO	Different foodborne pathogens	*A*. *kravanh* EO exhibited the best antibacterial activities against *B*. *subtilis* and *E*. *coli*.	[146]
*Anethum graveolens* L.	EO and acetone extract	*P. citrinum*, *A*. *niger*, *S. aureus*, *B*. *cereus*, *P*. *aeruginosa*	EO and extract showed different but both effective activities against tested microorganisms.	[147]
*Anethum graveolens* L.	diethyl-ether extract	*P*. *aeruginosa*, *E*. *coli*, *K*. *pneumoniae*, *M*. *luteus*, *E*. *faecalis*, *B*. *megaterium*, *S. aureus*	*A*. *graveolens* extract affected all of the bacteria tested.	[148]
*Anethum graveolens* L.	EO	*A*. *flavus*	*A*. *graveolens* EO is the most effective against aflatoxin production.	[117]
*Brassica jancea*	EO	*Vibrio parahaemolyticus* and *Vibrio vulnificus*	*B*. *jancea* EO could inhibit *V*. *parahaemolyticus* and *Vibrio vulnificus* inoculated sliced raw flatfish at 5 °C of storage.	[110]
*Brassica jancea*	Water extract	*E*. *coli*, *S. aureus*, *B*. *cereus*	*B*. *jancea* extract showed good inhibitory action at 1% concentration.	[149]
*Bunium persicum*	Volatile compounds	*F. oxysporum*	*B*. *persicum* showed the strongest effect compared with other 51 spices and herbs.	[150]
*Caesulia axillaris* Roxb.	EO	*A*. *flavus*	*C*. *axillaris* EO showed complete inhibition against *A*. *flavus* at 1.0 μg/mL.	[151]
*Capsicum froutescens*	Ethanol extract	*S. aureus*	*C*. *froutescens* extract showed the highest activity.	[152]
*Capsicum frutescens* L.	*n*-hexane, chloroform, ethyl acetate, acetone, and methanol extracts of dried seeds	*B*. *cereus*, *S. aureus*, MRSA, *E*. *coli*, *S. typhimurium*, *P*. *aeruginosa*, *K*. *pneumoniae*, *P. vulgaris*, *C*. *albicans*, *C*. *krusei*	Microwave assisted solvent extracts showed significant activities and *n*-hexane extract was effective against *P*. *aeruginosa* and *C*. *albicans*, while ethyl acetate extract was effective against *C*. *krusei*.	[153]
*Carum capticum*	EO	*Corynebacterium diphtheriae*, *S. aureus*, *Staphylococcus haemolyticus*, *B*. *subtilis*, *P*. *aeruginosa*, *E*. *coli*, *Klebsiella* species, *P*. *vulgaris*	*C*. *capticum* was very effective against all tested bacteria.	[154]
*Carum copticum*	EO	*S. aureus*, *B*. *cereus*, *E*. *coli*, *S*. *enteritidis*, *L*. *monocytogenes*	*C*. *copticum* EO was the most effective against tested bacteria with MICs of 0.03–0.5 mg/mL compared with two other spices.	[155]
*Cinnamomum burmannii*	Methanol crude extract	*B*. *cereus*, *L*. *monocytogenes*, *S. aureus*, *E*. *coli*, *Salmonella anatum*	MIC and MBC for *B*. *cereus* were 625 and 2500 μg/mL respectively, for four other bacteria were more than 2500 μg/mL.	[156]
*Cinnamomum cassia*	Ultra-fine powder	*E*. *coli*, *S. aureus*, *P*. *fluorescens*, *L. rhamnosus*, *B. thermosphacta*	*C*. *cassia* powder significantly reduced the microorganisms tested at the concentration ≤2.5% *w*/*v* and the inhibitory effects were positive correlated with concentrations.	[19]
*Cinnamomum tamala*	Leaves EO	*C*. *albicans*, *A*. *niger*, *A*. *fumigatus*, *R*. *stolonifer*, *Penicillium spp*.	The MFCs of EO against all the tested fungi were 230 μg/mL.	[157]
*Cinnamomum verum*	Bark and leaf extracts and EO	Bacteria isolated from urine samples, and *A*. *niger*	*C*. *verum* oil possessed stronger antimicrobial activities than extracts. *A*. *niger* showed no growth in the presence of oil.	[158]
*Cinnamomum verum*	EO	*E*. *coli*, *S. typhimurium*, *S. aureus*, *B*. *subtilis*, *A*. *flavus*, *C*. *albicans*	*C*. *verum* EO treated group showed significant decrease in viable bacterial counts.	[159]
*Cinnamomum verum*	EO	*S*. *typhimurium*, *S. paratyphi*, *E*. *coli*, *S. aureus*, *P*. *fluorescens*, *B. licheniformis*	*C*. *verum* bark EO showed the best antibacterial activities with mean MICs ranging from 2.9 to 4.8 mg/mL.	[160]
*Citrus aurantium* L.	Ethanol extract	*E*. *coli*, *P*. *aeruginosa*, *S. aureus*, *B. cereus*	*C*. *aurantium* showed strong antimicrobial activities against tested bacteria.	[161]
*Clinopodium ascendens*	EO	*S. aureus*, *S*. *faecium*, *S*. *mutans*, *Agrobacterium tumefasciens*, *E*. *coli*, *B. cinerea*, *C*. *albicans*	*C*. *ascendens* exhibited remarkable activity against *E*. *coli* and was active against *A*. *tumefasciens*, *S. aureus*, and *B*. *cinerea*.	[162]
*Corydothymus capitatus*	EO	*P*. *putida*	*C*. *capitatus* EO was the most active with a MIC of 0.025% *w*/*v* and a MTC of 0.006% *w*/*v*.	[163]
*Cotoneaster nummularioides*	Leaves EO	*B*. *cereus*, *S. aureus*, *Salmonella entrica*, *E*. *coli*	The extract of *C*. *nummularioides* showed strong effects on two Gram-positive microorganisms tested with higher sensitivity for *B*. *cereus* (MIC: 3.125 mg/mL).	[164]
*Croton hirtus*	EO	*E*. *coli*, *S. aureus*	*C*. *hirtus* EO was effective against *S. aureus* with MIC of 512 μg/mL.	[165]
*Cuminum nigrum* L.	Polyphenolic compounds	*B*. *subtilis*, *B*. *cereus*, *Enterobacter* spp., *E*. *coli*, *L*. *monocytogenes*, *S. aureus*, *Y. enterocolitica*	*C*. *nigrum* extract possessed significantly inhibitory effects on *B*. *subtilis*, *B*. *cereus*, and *S. aureus*.	[166]
*Curcuma longa*	Curcumin	*S. aureus*	Antibacterial activity of curcumin against *S. aureus* was enhanced with the increase of the concentration.	[167]
*Cunila galioides*	EO from aerial parts	15 bacterial species including *Bacillus* sp., *L*. *monocytogenes*, *S. aureus*, *A*. *hydrophila*, *E*. *faecalis* etc.	The oil of *C*. *galioides* citral efficiently controlled some microorganisms, showing both contact and gaseous activity.	[168]
*Dichrostachys glomerata*	Methanol extract	*Providencia stuartii*, *P*. *aeruginosa*, *K*.*pneumoniae*, *E*. *coli*, *E*. *aerogenes*, *E*. *cloacae*	*D*. *glomerata* extract inhibited the growth of all the 29 tested bacteria with MICs ≤ 1024 μg/mL.	[169]
*Echinops giganteus*	Methanol extract	*Mycobacterium tuberculosis* H(37)Rv, *Mycobacterium tuberculosis* H37Ra	The extract of *E*. *giganteus* was the most effective with MICs of 32 μg/mL and 16 μg/mL, respectively against H37Ra and H(37)Rv, compared with other 19 spices.	[170]
*Elettaria cardamomum*	Ethanol extract	4 strains of Gram-positive bacteria and 12 strains of Gram-negative bacteria	*E*. *cardamomum* extract was effective against a majority of the pathogens, MICs ranged from 9.4 to 18.75 mg/mL except *E. coli*, *B. cereus*, and *E. cloacae* which had a great sensitivity to the spice extract (MICs < 2.34 mg/mL).	[171]
*Elettaria cardamomum*	EO and various oleoresins	*S. aureus*, *B*. *cereus*, *E*. *coli*, *S. typhimurium*, *A*. *terreus*, *Penicillium purpurogenum*, *F. graminearum*, *Penicillium madriti*	The EO showed strong effects against bacteria tested at 3000 ppm, and the methanol and ethanol oleoresins gave the best results against *A*. *terreus* at 3000 ppm.	[172]
*Eucalyptus globulus*	Hydrodistillated extract	*S. aureus*, *B*. *subtilis*, *L*. *innocua*, *E*. *coli*, *P*. *aeruginosa*	*E*. *globulus* extract showed an inhibition effects against all the tested bacteria with MIC of 3 and 4 mg/mL.	[173]
*Eucalyptus largiflorens*	EO	*A*. *flavus*, *A*. *parasiticus*, *A*. *niger*, *Penicillium chryzogenum*, *P*. *citrinum*	The leaf oil of *E*. *largiflorens* showed higher antifungal activities than four other *Eucalyptus* spices.	[174]
*Eucalyptus radiata*	EO	*P*. *aeruginosa*, *E*. *coli* , *K*. *pneumoniae*, *S*. *typhimurium*, *Acinetobacter baumannii*, *P*. *aeruginosa*, *K*. *pneumoniae*	*E*. *radiate* showed better antibacterial activities with MICs ranging from 8 to 32μL/mL.	[175]
*Eugenia caryophyllum* Bullock and Harrison	Aqueous extract	*S. aureus*, *S*. *typhimurium*, *E*. *coli*, *S*. *epidermidis*, *L*. *plantarum*, *P*. *vulgaris*	The MICs and MBCs against all tested bacteria ranged from 1 to 4 g/L and 2 to 8 g/L, respectively.	[176]
*Foeniculum vulgare* ssp. piperitum	EO	*A. alternate*, *F. oxysporum*, *R. solani*	100% fungistatic effects were observed with 40 ppm doses of *F*. *vulgare* oils.	[177]
*Glaucium elegans*	Methanol extract	*E*. *coli*, *S. aureus*, *S*. *enteritidis*, *Bacillus anthracis*, *Proteus*	*G*. *elegans* methanol extract had significant antibacterial effects.	[178]
*Gloriosa superba Linn*	Methanol extract and fractions in different solvent systems	*C*. *albicans*, *Candida glaberata*, *Trichophyton longifusus*, *M. canis*, *S*. *aureus*, *E*. *coli*, *B*. *subtilis*, *K*. *pneumonae*, *S*. *flexneri*, *S*. *typhimurium*	The *n*-butanol fraction of *G*. *superba* showed excellent antifungal activities and chloroform fraction showed the highest antibacterial activity against *S*. *aureus*.	[179]
*Helichrysum* species	Methanol extracts	13 bacteria and 2 yeasts	All the extracts showed significant antimicrobial activities against all tested microorganisms.	[180]
*4 Helichrysum* Mill. plants	Methanol extracts	*A. hydrophila*, *Bacillus brevis*, *B*. *cereus*, *K*. *pneumoniae*, *P*. *aeruginosa*, *S. aureus*, *E*. *coli*, *M. morganii*, *M. smegmatis*, *P. mirabilis*, *Y. enterocolitica*, *S*. *cerevisiae*	The methanol extracts had antibacterial activities against the first six microorganisms listed.	[181]
horseradish	Aqueous extract	*S. aureus*	Horseradish water extract showed a higher biological activity.	[182]
*Hyssopus officinalis* L.	EO	*A*. *niger*, *A*. *ochraceus*, *A*. *versicolor*, *A*. *fumigatus*, *Cladosporium cladosporioides*, *Cladosporium fulvum*, *Penicillium funiculosum*, *Penicillium ochrochloron*, *Trichoderma viride*, *C*. *albicans*	All tested EO and deodorized extracts showed activities with the MICs ranging from 4 to 16 mg/mL.	[183]
*Laser trilobum* L.	Methanol extract	*S. aureus*, *P. vulgaris*, *P. mirabilis*, *B*. *cereus*, *A. hydrophila*, *E*. *faecalis*, *K*. *pneumoniae*, *S*. *typhimurium*, *E*. *aerogenes*, *E*. *coli*	The fruit extract had significant antimicrobial effects on pathogen bacteria.	[184]
*Laurus nobilis*	Ethanol extract	4 Gram-positive bacteria and 12 Gram-negative bacteria	*L*. *nobilis* extract was effective in inhibiting a majority of the pathogens, MICs ranged from 4.7 to 9.4 mg/mL.	[185]
*Laurus nobilis* L.	EO and leaves ethanol, water and hot water extract	*B*. *thermosphacta*, *E*. *coli*, *L*. *innocua*, *L*. *monocytogenes*, *P*. *putida*, *S*. *typhimurium*, *Shewanella putrefaciens*	*L*. *nobilis* EO exhibited strong antibacterial activities against all tested bacteria.	[186]
*Laurus nobilis* L.	Aqueous, ethanol, ethyl acetate and hexane extracts	*B*. *cereus*, *S. aureus*, *E*. *coli*, *K*. *pneumoniae*, *C*. *albicans*	Only aqueous extract of *L*. *nobilis* showed anticandidal activities among the tested 8 plants.	[187]
*Lavandula officinalis*	EO	*L*. *innocua* and *P*. *fluorescens*	*L*. *officinalis* EO showed the highest activity against *L*. *innocua*.	[188]
*Lichen Xanthoria parietina*	Acetone extract	*S. aureus*, *E*. *faecalis*, *P*. *vulgaris*, *P*. *mirabilis*, *S*. *typhimurium*, *E*. *cloacae*, *E*. *aerogenes*, *P*. *aeruginosa*, *K*. *pneumoniae*, *R. solani*, *Botridis cinerea*, *C*. *albicans*	*X*. *parietina* acetone extract and parietin showed similar activities on the nine bacteria tested, but less active than parietin on the three fungi tested.	[189]
*Lippia grandis* Schauer.	EO	*E*. *coli*, *P*. *aeruginosa*, *K*. *pneumoniae*, *S. aureus*, *E*. *faecalis*	The EO was effective against 75% of the microorganisms analyzed especially *S. aureus*, *E*. *faecalis*, and *E*. *coli*.	[190]
*Lippia javanica*	Acetone and aqueous extracts	*S. aureus*, *L*. *monocytogenes*, *S*. *typhimurium*, *E*. *coli*, *A*. *fumigatus*, *A*. *niger*, *M. canis*, *Microsporum gypseum*, *T. tonsurans*, *T*. *rubrum*, *T*. *mucoides*, *Penicillium aurantiogriseum*, *Penicillium chrysogenum*	The aqueous and acetone extracts were active against the bacterial strains, and the acetone extract exhibited the antifungal activities higher than even the reference drugs.	[191]
*Lippia origanoides* H.B.K.	EO	*C*. *albicans*, *Candida parapsilosis*, *Candida guilliermondii*, *Cryptococcus neoformans*, *Trichophyton rubrum*, *Fonsecaea pedrosoi*, *S. aureus*, *Lactobacillus casei*, *S*. *mutans*	*L*. *origanoides* EO showed highly significant inhibition zones for all microorganisms tested.	[192]
*Litsea cubeba*	EO	*E*. *coli*	The MIC and MBC of *L*. *cubeba* against *E*. *coli* were both 0.125% *v*/*v*.	[193]
*Melissa officinalis* L.	Ethanol, ethyl acetate and aqueous extracts	*Agrobacterium tumefaciens*, *Bacillus mycoides*, *B*. *subtilis*, *E. cloaceae*, *Erwinia carotovora*, *E*. *coli*, *Proteus* sp., *P*. *fluorescens*, *S. aureus*	*M. officinalis* ethanol, ethyl acetate, and aqueous extracts significantly enhanced the effectiveness of tested preservatives (sodium benzoate, sodium nitrite, and potassium sorbate).	[194]
*Mentha piperita L*.	EO	*T. rubrum*, *T*. *tonsurans*, *T*. *schoenleinii*, *T*. *mentagrophytes*, *M. canis*, *M*. *fulvum*	For effective concentration of *M*. *piperita* oil against tested antropophilic dermatophytes, and MICs ranged from 0.1 to 1.5 μL/mL.	[195]
*Mentha spicata* L.	hexane, chloroform, ethyl acetate, and aqueous fractions of ethanol extract	*Salmonella paratyphi*, *Shigella boydii*, *S. aureus*, *E*. *coli*, *Vibrio cholera*, *P*. *aeruginosa*, *E*. *faecalis*, *S*. *typhimurium*, *P*. *vulgaris*, *K*. *pneumoniae*	*M*. *spicata* ethanol extract and its solvent fractions effectively inhibited half of the microorganism growth.	[196]
*Myristica argentea*	Water extract	*E*. *coli* and *S. aureus*	*M*. *argentea* were more effective against *E*. *coli* (MIC of 9.80 mg/mL) and *S. aureus* (MIC of 6.20 mg/mL).	[197]
*Myristica fragrans*	-	20 different serogroups of *E*. *coli*, 8 serotypes of *Salmonella*, *L*. *monocytogenes*, *A. hydrophila*	*M. fragrans* showed good anti-listerial activity, although activities against *E*. *coli* and *Salmonella* were serotype dependent.	[103]
*Myristica fragrans*	Ethyl acetate and ethanol extracts of flesh, mace and seed	*S*. *mutans*, *Streptococcus mitis*, *Streptococcus salivarius*, *Aggregatibacter actinomycetemcomitans*, *P. gingivalis*, *Fusobacterium nucleatum*	Flesh ethyl acetate extract had the highest effects against tested bacteria with mean MICs ranging from 0.625 to 1.25 mg/mL among all tested extracts.	[198]
*Myrtus communis*	EO	*P*. *aeruginosa*, *S*. *typhimurium*, *E*. *coli*, *A. hydrophila*, *L*. *monocytogenes*, *C*. *albicans*	*M*. *communis* EO exhibited antimicrobial activities against all tested microorganisms, especially Gram-negative bacteria.	[199]
*Myrtus communis* L.	Methanol, ethyl acetate, acetone extracts	*S. aureus*, *P*. *vulgaris*, *P*. *mirabilis*	The most effective extract was the methanol extract from *M*. *communis* leaves against *S. aureus*.	[200]
*Myrica gale* L.	EO	*A*. *flavus*, *Cladosporium cladosporioides*, *Penicillium expansum*	A complete antifungal activity was observed at 1000 ppm of *M. gale* EO against *Cladosporium cladosporioides*.	[201]
*Nepeta alpina*	EO	*Bacillus pumilus*, *E*. *coli*, *Kocuria varians*, *L*. *monocytogenes*, *P*. *aeruginosa*, *S*. *typhimurium*, *A*. *niger*, *A*. *flavus*, *C*. *glabrata*	The EO was active against *L*. *monocytogenes* with MIC of 32 μg/mL.	[202]
*Nigella saliva* L.	Aqueous extracts	*Uromyces appendiculatus*	*N*. *saliva* extract was effective against *U. appendiculatus* and controlled rust similar to mancozeb fungicide at 2 and 3% concentrations.	[203]
*Nigella sativa* L.	n-hexan extract	24 pathogenic, spoilage and lactic acid bacteria	*N*. *sativa* oil showed antibacterial activities against all the bacteria at all concentrations (0.5%, 1.0% and 2.0%) tested.	[204]
*Ocimum canum*	EO	*B*. *subtilis*, *E*. *coli*, *K. pneumoniae*, *M. luteus*, *P*. *aeruginosa*, *Raoultella planticola*, *S*. *typhimurium*, *S. mutans*	MICs of *O. canum* ranged from 0.43 to 2.08 μL/mL against 7 out of 10 bacteria tested.	[205]
*Ocimum gratissimum* L.	EO	*A*. *flavus*, *A*. *niger*, *Aspergillus fumigatus*, *Aspergillus terreus*, *Aspergillus sydowi*, *Aspergillus alternate*, *Penicillium italicum*, *Fusarium nivale*, *C. lunata*, *Cladosporium* spp.	The EO exhibited antifungal activities against fungal isolates from some spices and showed better efficacy as fungi toxicant than prevalent fungicide Wettasul-80.	[206]
*Ocimum sanctum*	EO	*A*. *flavus*	MIC: 0.3 μL/mL.	[207]
*Ocimum sanctum* L.	EO	*A*. *flavus*, *Aspergillus fumigatus*, *Aspergillus clavatus*, *Aspergillus orizae S. aureus*, *E*. *faecalis*, *E*. *coli*, *enterohemorrhagic E*. *coli*, *P*. *aeruginosa*, *S*. *flexneri*	*O. sanctum* EO exhibited antimicrobial activities against all tested pathogens at concentrations of 0.125–32 μL/mL except *P*. *aeruginosa*.	[208]
*Ocimum suave*	EO	*S. aureus*, *S*. *epidermidis*, *S*. *mutans*, *S*. *viridans*, *E*. *coli*, *E*. *cloacae*, *K*. *pneumoniae*, *P*. *aeruginosa*, *C*. *albicans*, *C*. *tropicalis*, *C*. *glabrata*	*O*. *suave* EO showed the strongest antibacterial activities with MICs ranging from 0.05 to 1.37 mg/mL.	[209]
*Olea europaea* L.	Methanol extract	*S. aureus*, *S*. *epidermidis*, *S*. *pyogenes*, *Streptococcus agalactiae*, *S*. *enterica* serovar Typhi, *P*. *aeruginosa*, *Acetobacter calcoaceticus*, *C*. *albicans*, *P*. *vulgaris*, *S*. *faecalis*, *S*. *dysenteriae*, *K*. *pneumoniae*, *E*. *coli*, *V*. *cholera*, *C. xerosis*	*O*. *europaea* methanol extract showed strong antibacterial activities against *S. aureus*, *S*. *epidermidis*, and *S*. *pyogenes* at MICs range of 31.25–62.5 μg/mL.	[210]
*Origanum marjorana*	Water extract	*Vibrio parahaemolyticus*	*O*. *marjorana* showed the lowest MICs against *V*. *parahaemolyticus* both in a nutrient rich and poor medium.	[211]
*Origanum minutiflorum*	EO	*E*. *coli*, *S. aureus*, *S*. *enteritidis*, *L*. *monocytogenes*, *L*. *plantarum*	Whey protein based edible films incorporated with *O*. *minutiflorum* EO was the most effective at 2% level.	[212]
*Orthosiphon stamineus* Benth.	Methanol and aqueous extracts	*V*. *parahaemolyticus*	*V*. *parahaemolyticus* was more susceptible to 50–100% methanol extracts of *O*. *stamineus*.	[213]
*Peganum harmala* L.	Methanol extract	*S. aureus*, *S*. *epidermidis*, *S*. *pyogenes*, *S*. *agalactiae*, *S*. *enterica* serovar Typhi, *P*. *aeruginosa*, *Acetobacter calcoaceticus*, *C*. *albicans*, *P*. *vulgaris*, *S*. *faecalis*, *S*. *dysenteriae*, *K*. *pneumoniae*, *E*. *coli*, *V*. *cholera*, *C. xerosis*	*P*. *harmala* seed showed MICs of 31.25–62.5, 250, 125–250, and 31.25–250 μg/mL, respectively for *S. aureus*, *S*. *enterica serovar* Typhi, *Acetobacter calcoaceticus*, and *C*. *albicans*.	[210]
*Pimenta dioica* L.	Alcoholic and hexane extracts	*P*. *fluorescens*, *B. megaterium*, *A*. *niger*, *Penicillium* sp.	Alcoholic and hexane extracts of *P*. *dioica* exerted significant inhibitory effects on both the bacteria and fungi.	[214]
*Pimpinella anisum* L.	EO of fruit	*A. alternate*, *A*. *niger*, *A*. *parasiticus*	The most sensitive fungus for *P*. *anisum* oil was *A*. *parasiticus*.	[215]
*Pimpinella anisum* L.	EO	16 microorganisms	*P*. *anisum* EO exhibited strong antifungal activities against *R. glutinis*, *A. ochraceus*, and *F. moniliforme*.	[78]
*Pimpinella anisum* L.	EO	*C*. *lipolytica*, *H. uvarum*, *Pichia membranaefaciens*, *R. glutinis*, *S. pombe*, *Z. rouxii*, *A*. *flavus*, *A*. *ochraceus*, *A*. *parasiticus*, *F. moniliforme*	*P*. *anisum* EO completely inhibited the growth of tested fungi.	[78]
*Piper capense*	EO	*S. aureus*, *E*. *faecalis*, *C*. *albicans*	*P*. *capense* showed moderate activities against tested microorganisms.	[216]
*Piper guineense*	powder	*B*. *cereus*, *Bacillus coagulans*, *B*. *enterobacter* sp., *A*. *niger*, *R*. *stolonifer*	*P*. *guineense* inhibited *R*. *stolonifer* at concentrations above 0.5%.	[217]
*Phlomis oppositiflora*	Methanol, ethanol, ethyl acetate extracts and EO	*E*. *coli*, *S. aureus*, *K*. *pneumonia*, *M. smegmatis*, *P*. *aeruginosa*, *E*. *cloacae*, *B*. *megaterium*, *M. luteus*, *R. rubra*, *C*. *albicans*, *K. marxianus*	*P*. *oppositiflora* contains antimicrobial components against various microorganisms.	[218]
*Ramalina* species	Acetone, methanol and ethanol extracts	*E*. *coli* and *S. aureus*	The MICs of all extracts ranged from 64 to 512 g/mL for all bacterial strains tested.	[219]
*Rhus coriaria* L.	80% (*v*/*v*) aqueous alcohol extract	*S. aureus*, *B*. *cereus*, *E*. *coli*, *S*. *typhimurium*, *P. vulgaris*, *S*. *flexneri*	The MICs of *R*. *coriaria* extract against the tested bacteria ranged from 0.04% to 0.2%.	[220]
*Rhus coriaria*	Water extract	*B*. *cereus*, *L*. *monocytogenes*, *E*. *coli*, *S*. *typhimurium*	*R*. *coriaria* extract was the most effective against the four bacteria tested.	[221]
*Salvia officinalis* L.	EO	13 bacterial strains and 6 fungi	Sage EO was more effective against *E*. *coli*, *S. typhimurium*, *S*. *enteritidis*, and *S. sonei*.	[92]
*Salvia officinalis* L. (sage)	80% ethanol extract	*Campylobacter coli*, *E*. *coli*, *Streptococcus infantis*, *B*. *cereus*, *L*. *monocytogenes*, *S. aureus*	Sage extract showed the best antibacterial activities compared with four other plants, especially against Gram-positive bacteria and *C*. *coli*.	[222]
*Salvia officinalis* L.	EO	*E*. *coli*, *P*. *aeruginosa*, *Enterobacter* sp., *S. aureus*	Microwave-EO of *S*. *officinalis* possessed good antibacterial activities than the hydrodistilled oil.	[223]
*Salvia leriifolia*	Methanol extract	*S. aureus*	*S*. *leriifolia* extract exhibited antimicrobial activity against *S. aureus*.	[224]
*Santolina chamaecyparissus* L.	EO	*K*. *pneumonia* and *C*. *albicans*	*S*. *chamaecyparissus* EO was very active against the two microorganisms listed.	[225]
*Satureja cuneifolia* Ten.	EO	*E*. *coli*, *Campylobacter jejuni*, *S*. *sonnei*, *S. aureus*, *L*. *monocytogenes*, *B*. *cereus*, *P*. *aeruginosa*, *S*. *enteritidis*	MICs of *S*. *cuneifolia* EO for tested bacteria were in the range of 600–1400 μg/mL.	[226]
*Satureja kitaibelii*	EO	30 pathogenic microorganisms	*S*. *kitaibelii* EO showed significant activities against foodborne microbes (MIC: 0.18–25.5 μg/mL), multiresistant bacterial isolates (MIC: 6.25–50.0 μg/mL), and dermatophyte strains (MIC: 12.5–50.0 μg/mL).	[227]
*Satureja wiedemanniana*	EO	37 *Bacillus* strains	Both *S*. *wiedemanniana* EO and its main component p-cymene exhibited strong antimicrobial activities against some *Bacillus* strains.	[228]
*Satureja* species	EOs	*A*. *niger*, *Penicillium digitatum*, *B. cinerea*, *R*. *stolonifer*	The EOs exhibited fungicidal activities against *P*. *digitatum*, *B*. *cinereal*, and *R*. *stolonifer*.	[229]
*Silene laxa*	Ethyl acetate, chloroform, methanol, ethanol and acetone extract	*P*. *aeruginosa*, *E*. *cloacae*, *B*. *megaterium*, *E*. *cloacae*, *S. aureus*	*S*. *laxa* leaves ethanol extract showed the best activities against *P*. *aeruginosa*, *E*. *cloacae*, *B*. *megaterium*, while the methanol extracts of *S. laxa* fruits showed the best antibacterial activity against *B.megaterium*.	[230]
Summer savory	-	*A*. *niger*, *A. alternate*, *A*. *parasiticus*	0.5% summer savory extract showed 100% inhibition till the seventh day of incubation.	[231]
*Syzygium aromaticum* L.	Water extract	*S. aureus*, *S*. *epidermidis*, *S*. *pyogenes*, *S*. *agalactiae*, *S*. *enterica* serovar Typhi, *P*. *aeruginosa*, *Acetobacter calcoaceticus*, *C*. *albicans*, *P*. *vulgaris*, *S*. *faecalis*, *S*. *dysenteriae*, *K*. *pneumoniae*, *E*. *coli*, *V*. *cholera*, *C. xerosis*	*S*. *aromaticum* water extract showed antibacterial activities with MICs in the range of 31.25–250 μg/mL for *S. aureus*, *S*. *epidermidis*, *S*. *pyogenes*, *S*. *enterica* serovar Typhi, *Acetobacter calcoaceticus*, and *P*. *aeruginosa*.	[210]
*Thymbra spicata* L.	Decoction	*F. oxysporum f*. sp. *phaseoli*, *M. phaseoli*, *B. cinerea*, *R*. *solani*, *A*. *solani*, *A*. *parasiticus*	*T*. *spicata* completely inhibited the mycelial growth of fungi and showed a complete fungicidal effect on molds.	[39]
*Thymus capitata*	EO	*L*. *monocytogenes*	MICs ranged from 0.32 to 20 mg/mL.	[232]
*Thymus capitatus*	EO	*L*. *innocua*, *S. marcescens*, *P*. *fragi*, *P*. *fluorescens*, *A. hydrophila*, *Shewanella putrefaciens*, *Achromobacter denitrificans*, *E*. *amnigenus*, *E*. *gergoviae*, *Alcaligenes faecalis*, *Leuconostoc carnosum*	*T*. *capitatus* EOs showed inhibitory effects on the 10 tested bacteria with MICs ranging from 1.87 to 7.5 μL/mL.	[233]
*Thymus cappadocicus* Boiss.	EO	13 bacteria and 2 yeasts	*T*. *cappadocicus* EO showed great antimicrobial activities against microorganisms tested.	[234]
*Thymus eigii*	EO	*M. luteus*, *B*. *megaterium*, *B*. *brevis*, *E*. *faecalis*, *P*. *pyocyaneus*, *M. smegmatis*, *E*. *coli*, *A. hydrophila*, *Y. enterocolitica*, *S. aureus*, *S*. *faecalis*, *S*. *cerevisiae*, *K. fragilis*	*T*. *eigii* EO showed the highest antimicrobial activities compared with two other plants.	[235]
*Thymus piperella*	EO	*L*. *innocua*, *S. marcescens* , *P*. *fragi*, *P*. *fluorescens*, *A. hydrophila*, *S. putrefaciens*, *A. denitrificans*, *E. amnigenus*, *E*. *gergoviae*, *A. faecalis*, *L. carnosum*	*T*. *piperella* EO had inhibitory effects on 5 of the 11 bacteria tested.	[236]
*Thymus serpyllum*	EO	*Penicillium* sp., *Alternaria* sp., *Aureobasidium* sp.	8 mg/disc EO of *T*. *serpyllum* has a good efficiency by inhibiting the germination of spores from 80% to 100%.	[237]
*Trachyspermum ammi* L.	EO	*A*. *niger*, *A*. *flavus*, *A*. *oryzae*, *A*. *ochraceus*, *F. monoliforme*, *F*. *graminearum*, *Pencillium citrium*, *P*. *viridicatum*, *P*. *madriti*, *C. lunata*	*T*. *ammi* EO exhibited a broad spectrum of fungi toxic behavior against all tested fungi.	[238]
*Xylopia aethiopica*	-	*Sclerotium rolfsii*	*X*. *aethiopica* extract was the most effective against *S*. *rolfsii* compared with four other spices.	[239]
*Zanthoxylum piperitum*	Polymeric procyanidin	*S. aureus*	A polymeric proanthocyanidin purified from the fruit of *Z*. *piperitum*, noticeably decreased the MICs of β-lactam antibiotics for MRSA.	[240]
*Zanthoxylum schinifolium*	EO	*S. aureus*, *S*. *epidermidis*, *B*. *subtilis*, *S*. *typhimurium*, *P*. *aeruginosa*, *S*. *dysenteriae*, *E*. *coli*	*Z*. *schinifolium* EO was particularly strong against *S*. *epidermidis*, with MIC 2.5 mg/mL.	[241]
*Zataria multiflora* Boiss.	80% (*v*/*v*) aqueous alcohol extract	*S. aureus*, *B*. *cereus*, *E*. *coli*, *S*. *typhimurium*, *P*. *vulgaris*, *S*. *flexneri*	The MICs of *Z*. *multiflora* against the tested bacteria ranged from 0.4% to 0.8%.	[220]
